# Nature-Inspired Strategies in Cancer Management: The Potential of Plant Extracts in Modulating Tumour Biology

**DOI:** 10.3390/ijms26146894

**Published:** 2025-07-18

**Authors:** Marcin Hołota, Małgorzata M. Posmyk

**Affiliations:** Department of Plant Ecophysiology, Faculty of Biology and Environmental Protection, University of Lodz, Banacha 12/16, 90-237 Lodz, Poland; malgorzata.posmyk@biol.uni.lodz.pl

**Keywords:** cancer, plant extracts, phytochemicals, proapoptotic activity, antiproliferative activity, antiangiogenic activity, immunomodulatory activity, revised hallmarks of cancer

## Abstract

Cancer is a serious group of diseases that is a huge problem on a global scale and is the second most common cause of death. Commonly used therapies do not always lead to the complete elimination of diseased cells or tissues and are also burdened with side effects that reduce the quality of life of patients. Due to these difficulties, new therapeutic approaches are still being sought. In recent years, there has been a return to interest in natural methods of treating various diseases, among which phytochemicals are particularly interesting. This article reviews plant extracts with anticancer properties with different mechanisms of action (proapoptotic, antiproliferative, antiangiogenic, immunomodulatory). In addition, plant extracts that reduce the side effects of chemotherapy and the limitations and prospects for the use of plant extracts in anticancer therapy are described. Our goal was to create an up-to-date information base that would encourage scientists to intensify research into supplementing targeted anticancer therapies with additional protective and preventive measures, in which natural mixtures of phytochemicals (plant extracts) are effective allies. At the same time, we encourage discussion on the limitations of their use in light of the orthodox principles of classical medicine and pharmacy (issues of safety, quality, drug purity, and dose precision), which are a priori correct but have not yet led to the elimination of cancer from the group of incurable diseases.

## 1. Introduction

Cancer is a group of diseases that constitutes a serious social and economic problem and poses a challenge to health care worldwide despite the enormous development of medicine. The number of newly diagnosed cases is constantly growing. Moreover, cancer is the second leading cause of death after cardiovascular system diseases. It is estimated that in 2020, 10 million deaths were caused by different cancers [1,2].

The cells that have undergone permanent genetic transformation, leading to their uncontrolled growth and division, are considered cancer cells. Unlike healthy cells, which function within a tightly regulated cell cycle, cancer cells lose this control, allowing them to proliferate without restriction. One of the main features of these cells is their independence from external growth signals and their ignoring of signals that inhibit division. This is most often due to mutations in so-called proto-oncogenes, e.g., *RAS* (rat sarcoma oncogene homolog; leads to continuous activation of growth signals, even without an external stimulus), *MYC* (avian myelocytomatosis oncogene homolog; its overexpression or amplification causes accelerated cell growth and impaired cell cycle control), and suppressor genes, such as *TP53* (encoding the p53 protein, which in response to DNA damage activates repair mechanisms, arrests the cell cycle, or induces apoptosis) or *RB1* (encoding the protein pRb, which inhibits the progression from G1 to S phase of the cell cycle by preventing division of cells with an unready genome), which in healthy cells act as “genome guardians” and prevent damaged cells from multiplying [3]. Cancer cells also demonstrate the ability to avoid apoptosis, or programmed cell death. Under normal conditions, apoptosis allows for the elimination of damaged or unnecessary cells, but in cancers, the mechanisms of this pathway are deactivated, often through mutations in genes encoding proteins such as Bcl-2, Bax, or caspases (described in Section 2). Thanks to this, cancer cells can survive despite serious genetic defects [4]. Another important feature of cancer cells is the ability to multiply without any limits. In healthy cells, the length of telomeres—the end sections of chromosomes—shortens with each cell division, which limits their number of divisions. In cancer cells, the enzyme telomerase is activated, which rebuilds telomeres, allowing for a theoretically infinite number of divisions. This is one of the key factors in the immortality of cancers [3]. In addition, cancer cells can stimulate angiogenesis, via various mechanisms process creating new blood vessels. By secreting different vascular endothelial growth factors (see Section 4), they create access to nutrients and oxygen, which allows the tumour to continue to grow. Without angiogenesis, the tumour would not be able to exceed a few millimetres in diameter. Another extremely dangerous feature of cancers is their ability to invade locally and create metastases. Cancer cells can penetrate surrounding tissues, enter blood or lymphatic vessels, and then colonise distant organs. This is a multi-stage process in which both changes in cell adhesion (including loss of E-cadherin expression) and activation of proteolytic enzymes (e.g., MMPs—extracellular matrix metalloproteinases) play a role, which enables them to overcome tissue barriers [5].

Cancer cells also exhibit genetic instability, which means that they are more susceptible to mutations and DNA damage. They often lose the ability to properly repair DNA, e.g., due to mutations in genes such as *BRCA1*, *BRCA2*, (breast cancer genes 1 and 2; essential for homologous recombination repair of DNA damage, chromatin remodelling, and maintaining genomic stability), *MLH1* (*MutL Homolog 1*; the human homolog of the bacterial MutL gene key in DNA mismatch repair pathway, it corrects base-pair mismatches that occur during DNA replication), which leads to the growth of further mutations and even greater tumour aggressiveness. Such genetic instability means that tumours are often heterogeneous—they contain many different cell clones, which makes their treatment difficult [3]. In addition to genetic changes, cancer cells are also characterised by an altered metabolism. In oxygenated conditions, healthy cells produce energy mainly through oxidative phosphorylation. Cancer cells, on the other hand, prefer anaerobic glycolysis (the so-called Warburg effect) when oxygen is available. Although this path is less energy efficient, it allows for rapid acquisition of substrates for the biosynthesis of essential cellular components [4]. Moreover, cancers demonstrate the ability to escape the surveillance of the immune system. Cancer cells can inhibit the activity of cytotoxic lymphocytes, reduce the expression of *MHC* (major histocompatibility complex, a group of genes that encode proteins on the cell surface that are essential for the immune system to recognise foreign molecules), and secrete immunosuppressive cytokines. Sometimes, so-called T-cell exhaustion also occurs, which weakens the immune response and allows the cancer to develop unnoticed [6].

The above-mentioned features/adaptations of cancer cells and their late detection mean that commonly used methods in cancer treatment are surgical resection of diseased tissues, chemotherapy, and radiotherapy [7]. However, these methods are not always effective and are also burdened with many side effects that negatively affect the life quality of patients [8]. The possibility of using a surgical procedure depends on the location of the tumour, the stage of the cancer, and the general condition of the patient. This is a serious intervention that places a heavy burden on the body and, unfortunately, carries a risk of not removing all cancerous foci. Despite the development of techniques used in radiotherapy, many patients still experience troublesome side effects that affect the tissues and organs subjected to radiation [9]. Also, drugs used in anticancer therapies attack normal cells in addition to cancer cells. As a result, chemotherapy is associated with side effects such as hepatotoxicity, nephrotoxicity, ototoxicity, cardiotoxicity, neurotoxicity, and secondary cancers [10,11,12]. The mentioned anticancer therapies’ weaknesses, limited effectiveness and frequent side effects, have activated the research of many scientists around the world are focused on limitation side effects or searching for new, safe therapeutic approaches [13].

Due to the renewed interest in natural medicine, more and more research is focused on the search for compounds of natural origin, and a particularly popular concept is considering plants as a source of compounds with high therapeutic potential. Although many anticancer drugs are of plant origin, it seems very likely that the possibility of using plants in anticancer therapy has not been exhausted. Plant extracts, which are rich in various bioactive compounds, have various effects important in terms of fighting cancer. Phytochemicals can inhibit cancer cell proliferation by affecting the cell cycle, including by stopping it in the G1 or G2/M phase. Many of them activate apoptosis pathways, both involving mitochondria (intrinsic pathway, e.g., by regulating Bcl-2/Bax proteins) and extrinsic pathways (by activating death receptors). Other phytochemicals limit angiogenesis by inhibiting the expression of VEGF (vascular endothelial growth factor) and the activity of metalloproteinases (MMPs), which hinders tumour growth and spread. In addition, some plant compounds (e.g., flavonoids) affect the expression of genes responsible for DNA repair and reduce oxidative stress, which may limit genetic instability. Their immunomodulatory properties are also important—some extracts stimulate the activity of NK cells and T lymphocytes and counteract tumour-induced immunosuppression. Furthermore, phytochemicals can inhibit metabolic pathways characteristic of cancer, for example, by inhibiting the activity of enzymes key to anaerobic glycolysis (Warburg effect) [14,15,16,17,18]. Because they involve proapoptotic, antiproliferative, and antiangiogenic effects, they can support the immune system and protect normal cells from the harmful effects of classic chemotherapy and even radiotherapy [19,20,21,22,23,24,25,26,27]. This article provides an overview of plant extracts that demonstrate anticancer activity via various mechanisms, as well as extracts that support the body during burdensome therapies.

## 2. Proapoptotic Mechanisms Induced by Plant Extracts

Apoptosis (or programmed cell death) is a physiological process essential/crucial mechanism for maintaining homeostasis at various stages of ontogenesis. It ensures proper embryogenesis, followed by the development and maturation of the organism, plays a role in regulating the immune system, and protecting the body from the proliferation/spread of abnormal cells [28,29,30]. Thus, it is a crucial defence mechanism against cancer development, leading to the elimination of damaged, mutated, or improperly functioning cells. This process can be initiated through two main pathways, which differ in their molecular mechanisms: the extrinsic (death receptor) pathway and the intrinsic (mitochondrial) pathway [31].

The extrinsic pathway is triggered by external signals mediated by death receptors belonging to the TNF (tumour necrosis factor) receptor family. The most notable receptors involved are Fas (CD95) and TNF-R1. Upon binding of specific ligands (e.g., FasL or TNF-α) to these receptors, a death-inducing signalling complex (DISC) is formed. DISC recruits and activates caspase-8, which is a key enzyme in this pathway. The activation of caspase-8 directly activates effector caspases, such as caspase-3, which are responsible for degrading intracellular proteins, ultimately leading to cell death. This pathway is rapid and relies on external signals to initiate apoptosis [32,33,34].

The intrinsic, or mitochondrial, pathway is regulated by intracellular stress signals, such as oxidative stress, DNA damage, or the absence of growth factors. A critical component of this pathway is the mitochondrion. Stress signals lead to the disruption of mitochondrial membrane integrity, causing the release of cytochrome c into the cytoplasm. In the cytoplasm, cytochrome c interacts with Apaf-1 (apoptotic protease-activating factor-1) and procaspase-9, forming a complex known as the apoptosome. This leads to the activation of caspase-9, which initiates the caspase cascade, ultimately activating effector caspases such as caspase-3, resulting in apoptosis. The intrinsic pathway is regulated by the Bcl-2 family of proteins, which can promote (e.g., Bax, Bak) or inhibit (e.g., Bcl-2, Bcl-xL) apoptosis, allowing the cell to respond appropriately to stress signals [35,36,37].

Although the two pathways can operate independently, there are points of interaction between them. For example, in certain cells, caspase-8 (extrinsic pathway) can activate the proapoptotic protein Bid, leading to the activation of the mitochondrial pathway, thus linking both mechanisms into a unified apoptotic cascade [38]. The regulation of apoptosis is multidirectional and depends on the signalling pathway. In turn, various types of proteins participate in the regulation of both pathways, including proapoptotic and antiapoptotic proteins. The balance between these two groups determines the fate of the cell, playing a critical role in both normal physiological processes and disease development [39].

Proapoptotic proteins promote apoptosis by disrupting mitochondrial membrane integrity and facilitating the release of apoptogenic factors, such as cytochrome c, which trigger the caspase cascade. The two main groups of proapoptotic proteins are BH3-only proteins and multidomain proapoptotic proteins [40]. BH3-only proteins are a subset of the Bcl-2 family and act as initiators of apoptosis in response to cellular stress signals such as DNA damage, oxidative stress, or growth factor deprivation. These proteins contain a single BH3 domain (Bcl-2 homology domain 3) that is essential for their proapoptotic function. Examples of BH3-only proteins include Bid (when cleaved by caspase-8—in the extrinsic pathway—truncated Bid (tBid) translocates to mitochondria, where it promotes mitochondrial outer membrane permeabilisation—MOMP), Bim, Puma, and Noxa (these proteins are activated by various stress signals and induce apoptosis by neutralising antiapoptotic proteins like Bcl-2 and Bcl-xL) [41]. Multidomain proapoptotic proteins contain multiple BH domains (BH1, BH2, and BH3) and are directly responsible for disrupting the mitochondrial membrane. The most well-known multidomain proapoptotic proteins are Bax (Bcl-2-associated X protein) and Bak (Bcl-2 homologous antagonist/killer). Upon activation by BH3-only proteins, Bax translocates from the cytosol to the mitochondria, where it inserts into the outer mitochondrial membrane and forms oligomeric pores. Bak is already located in the mitochondrial membrane and, when activated, similarly forms pores in the membrane. The oligomerisation of Bax and Bak results in MOMP, leading to the release of cytochrome c and other proapoptotic factors, which in turn activate caspase-9 and initiate the apoptotic cascade [42].

Two major families of antiapoptotic proteins are IAP (inhibitor of apoptosis proteins) and Bcl-2 family proteins. The IAP consists of proteins that directly inhibit the activity of caspases, the proteases responsible for executing apoptosis. Key members of this family include XIAP (X-linked IAP), c-IAP1, and c-IAP2. XIAP is one of the most potent inhibitors of apoptosis. It binds directly to active caspases, particularly caspase-3, caspase-7, and caspase-9, preventing their activity and halting the apoptotic process. c-IAP1 and c-IAP2 also have roles in regulating cell survival, but they do so through complex interactions with various signalling pathways, including those involved in the regulation of NF-κB (nuclear factor kappa-light-chain-enhancer of activated B cells; a family of transcription factor proteins, regulate the expression of many genes involved in immune response, inflammation, cell development pathways), which promotes cell survival. These IAP proteins often contain BIR (baculoviral IAP repeat) domains that mediate their interaction with caspases, effectively neutralising them. In addition to directly inhibiting caspases, some IAP proteins also function as ubiquitin ligases, tagging proapoptotic proteins for degradation, thereby further preventing apoptosis [43]. The Bcl-2 family of proteins is crucial for regulating the intrinsic (mitochondrial) pathway of apoptosis. This family includes both proapoptotic and antiapoptotic members, with the balance between these opposing groups determining cell fate. The antiapoptotic members include, i.a., Bcl-2, Bcl-xL, and Mcl-1. Bcl-2 and Bcl-xL are located on the outer mitochondrial membrane, and they both prevent the activation and oligomerisation of Bax and Bak, thus preserving mitochondrial integrity. By doing so, they inhibit the release of cytochrome c and other proapoptotic factors from the mitochondria into the cytoplasm, which would otherwise trigger the apoptosome formation and subsequent caspase activation [44]. Another antiapoptotic member—Mcl-1—also sequesters proapoptotic proteins, particularly Bak, to prevent mitochondrial permeabilisation. It is tightly regulated by post-translational mechanisms, such as phosphorylation and ubiquitination, ensuring rapid response to cellular stress [45]. Antiapoptotic proteins are regulated by cellular signals that promote survival, such as growth factors and cytokines. Their activity is essential for cells to resist apoptosis under normal physiological conditions or in response to mild stress. However, overexpression of antiapoptotic proteins, particularly Bcl-2 and Bcl-xL, is commonly associated with cancer, as it enables cells to evade apoptosis, leading to unchecked proliferation [44,46,47,48].

A very common direction of research on anticancer therapy is the search for apoptosis-inducing agents that would be safer for healthy cells. There are reports in the literature on the proapoptotic activity of natural compounds, with particular emphasis on phytochemicals contained in plant extracts (e.g., flavonoids, alkaloids, and terpenoids). Studies by Gregoriou et al. [49] suggest that extracts obtained from Cypriot carob (*Ceratonia siliqua* L.) pods exhibit anticancer activity against breast cancer cells while having no effect on healthy cells. The extracts obtained using diethyl ether and ethyl acetate were the most effective in inducing the intrinsic apoptosis pathway (by decreasing Bcl-2 proteins and activating the caspase-dependent apoptotic pathway) and inhibiting proliferation (by reducing serine phosphorylation at AKT Ser478, which increases the levels of p21 and p27, cell cycle inhibitors) selectively in MCF-7 breast cancer cells. The predominant polyphenols in these extracts were myricetin, naringenin, and kaempferol, which have previously been described as compounds with anticancer and health-promoting properties. Also, in Ayache’s [50] studies on various aqueous extracts of *Ceratonia siliqua* seeds, their anticancer properties were demonstrated. The seed extract was particularly effective against colon cancer cells, where over 40% of apoptotic cells were observed after treatment with the extract at a concentration of 1000 µg/mL. Chloroform extract from *Cucurbita ficifolia* fruit, due to modifying the activity of proteins: p53 (tumour suppressor protein responsible for monitoring and controlling the genetic integrity of cells), FAS (cell surface death receptor), FADD (FAS-associated death domain; apoptosis signal mediator), Bax and Bak, and subsequent modulation of caspase-3, caspase-8, and caspase-9 genes expression levels, showed an apoptosis-inducing effect in MCF-7 breast cancer cells. However, these extracts also showed a negative effect on normal hepatocytes, against which the cytotoxicity of the extracts was slightly lower [51]. Ethanolic extract of *Phoenix dactylifera* seeds showed an apoptosis-inducing effect in MDA-MB-231 triple-negative breast cancer cells. The authors associate this effect with the synergistic effect of bioactive factors, primarily rutin and quercetin [52]. Curcumin, derived from *Curcuma longa,* is another plant extract with well-documented proapoptotic properties. Curcumin can modulate various signalling molecules involved in apoptosis, including p53, Bax, Bcl-2, and caspase-3, promoting cancer cell death while sparing normal cells. Studies show that curcumin induces apoptosis in colorectal, breast, and prostate cancer cell lines [53]. Methanol extracts of *Phyllanthus muellerianus* and *Ficus exasperata* showed a proapoptotic effect via the Bax/Cytochrome C/Caspase 3-9 signalling pathway against PC-3 prostate cancer cells [54]. Aqueous extracts of *Elaeagnus angustifolia* flowers induced apoptosis in two triple-negative breast cancer lines (MDA-MB-231 and MDA-MB-436) by affecting the expression of proapoptotic and antiapoptotic genes. The advantage is that these extracts did not negatively affect the non-cancerous epithelial cell line (MCF 10A) [55]. Ethanol extract from *Annona cherimola* leaves triggered the mitochondrial apoptosis pathway in triple-negative breast cancer cells (MDA-MB-231). Studies showed the release of cytochrome C, an increase in p21 (cell cycle suppressor protein) levels, and an increase in the Bax/Bcl-2 ratio [56]. Studies of this extract on acute myeloid leukaemia lines showed similar activity, which is based on activation of caspases 8 and 9, and an increase in the Bax/Bcl-2 ratio. Furthermore, in this case, PARP (Poly(ADP-ribose)-polymerase) cleavage was also observed, which impairs the ability of cancer cells to repair their DNA [57]. Ethanolic extracts of *Ipomoea carnea* leaf powder at a concentration of 20 µg/mL showed proapoptotic activity against breast cancer cells after 24 h of incubation [58]. Methanol extract of *Allium atroviolaceum* flowers triggered apoptosis of two breast cancer cell lines, MCF-7 and MDA-MB-231, by both extrinsic and intrinsic pathways. Studies showed that apoptosis induced by the extract is caspase- and Bcl-2-dependent and p53 transcription-independent [59]. Methanol extract of *Avicennia marina* leaves induced apoptosis of MDA-MB 231 cells by causing their DNA fragmentation, increased p53 expression, and decreased Bcl2 protein levels [60]. Methanol extract of *Holarrhena floribunda* showed an apoptosis-triggering effect in HeLa, MCF-7, and HT-29 cells. In the case of HeLa lines, an increase in caspase 3 activity was observed, while in the case of MCF-7 and HT-29 lines, its activity was decreased. Caspase-9 activity decreased concentration-dependently in all three cell lines [61]. *Withania somnifera* (Ashwagandha) has been extensively studied for its proapoptotic effects on cancer cells. Active compounds from this plant, such as withaferin A, have been shown to induce apoptosis by activating both the intrinsic and extrinsic apoptotic pathways. Specifically, withaferin A leads to mitochondrial dysfunction and increases the activity of caspases, the enzymes responsible for executing apoptosis [62].

Apoptosis, as a natural mechanism of eliminating damaged and cancerous cells, is a key target in anticancer therapy. Numerous studies have demonstrated that natural plant extracts containing bioactive compounds (such as flavonoids, alkaloids, terpenoids) can effectively activate apoptotic pathways—both internal (mitochondrial) and external (dependent on death receptors)—in cancer cells. Many plant extracts (e.g., from *Ceratonia siliqua*, *Curcuma longa*, *Phoenix dactylifera*, *Annona cherimola*, and *Withania somnifera*) exhibit selective cytotoxicity against cancer cells while sparing healthy ones, making them a promising therapeutic option when used alongside conventional drugs. The ethyl acetate extract obtained from the pulp of ripe *Ceratonia siliqua* (carob) significantly reduced the viability of MCF-7 breast cancer cells at concentrations starting from 0.5 mg/mL, while showing no significant effect on the normal MCF-10A cell line. The IC_50_ value for MCF-7 was 0.27 ± 0.03 mg/mL, whereas MCF-10A cell viability did not decrease to 50% even at higher concentrations. Similarly, the diethyl ether extract from ripe carob pulp also demonstrated selective cytotoxicity toward the MCF-7 cell line, with an IC_50_ of 0.96 ± 0.11 mg/mL compared to 2.34 ± 0.03 mg/mL for MCF-10A cells [50]. The ethanolic extract of *C. siliqua* leaves exhibited significant cytotoxicity against various cancer cell lines in a dose-dependent manner, with MCF-7 cells being the most sensitive. The extract did not exhibit cytotoxic effects on normal peripheral blood mononuclear cells (PBMCs). Specifically, the IC_50_ for MCF-7 cells was 32.44 ± 5.23 µg/mL, while for MDA-MB-231 and MDA-MB-436 cells, the IC_50_ values were 40.05 ± 3.21 µg/mL and 53.55 ± 5.35 µg/mL, respectively. For PBMCs, the IC_50_ exceeded 890 µg/mL, indicating minimal cytotoxicity [63]. Data from MTT assays showed that the ethanolic extract from *Phoenix dactylifera* (date palm) seeds decreased the viability of MDA-MB-231 cells to 91.9%, 87.8%, 74.3%, 61.7%, and 48.4% at concentrations of 10, 25, 50, 75, and 100 μg/mL, respectively, after 24 h. After 48 h, a more pronounced cytotoxic effect was observed, reducing cell viability to 90.5%, 84%, 67.6%, 54.9%, and 43.6%, respectively. The IC_50_ values of the extract were 101.6 μg/mL and 85.86 μg/mL after 24 and 48 h, respectively. Similar results were obtained for MCF-7 cells, where viability decreased to 96.4%, 90.9%, 82.3%, 69.6%, and 51% after 24 h, and to 92.8%, 86.5%, 77%, 61.3%, and 46.4% after 48 h of incubation. In HepG2 liver cancer cells, viability declined to 96.1%, 92.3%, 84.3%, 75.1%, and 62.6% at 24 h, and to 94.5%, 90.8%, 81.1%, 71.1%, and 55.1% at 48 h, across the same concentration range. Importantly, the extract exhibited no significant cytotoxic effect on normal Vero cells, with viability remaining above 93% at all tested concentrations and time points [52]. Furthermore, the ethanolic seed extract of *Annona cherimola* demonstrated cytotoxic activity against AGS gastric cancer cells, with an IC_50_ of 80.43 ± 3.92 μg/mL after 48 h. For the normal gastric cell line GES-1, the IC_50_ was higher, at 113.13 ± 13.10 μg/mL, indicating selective activity [64]. Finally, methanolic extracts from the leaves and stems of *Withania somnifera* showed notable cytotoxicity in HepG2 liver cancer cells, with IC_50_ values of 43.06 ± 0.615 μg/mL and 45.60 ± 0.3 μg/mL, respectively. In contrast, for the non-cancerous L929 cell line, the IC_50_ values were higher—78.77 ± 0.795 μg/mL for the leaf extract and 90.55 ± 0.800 μg/mL for the stem extract—suggesting a degree of selectivity [65].

Undoubtedly, plant extracts can support traditional chemotherapy by enhancing cancer cell apoptosis. Further research, standardisation, and safety assessment may pave the way for their broader use as effective and less toxic agents in supportive anticancer therapies.

## 3. Suppression of Cancer Cell Proliferation by Phytochemicals

Although cancer cells are most often structurally and functionally like the cells from which they originate, in the process of neoplastic transformation, they acquire characteristic features that distinguish them from normal cells. Neoplastic transformation is a process that occurs under the influence of genetic and environmental factors, because of which many damages to the genetic material accumulate. Most differentiated cells are at risk of losing the ability to control division and take on characteristics typical of cancer cells. These features allow them to form a cancerous tumour, and in the final stages of its development, to metastasise. Unlimited cell proliferation is one of the most important processes for the development of cancer. Loss of control over the cell cycle leads to uncontrolled divisions and quick growth of the cancer tumour. This is often associated with mutations of genes responsible for controlling the cell cycle, including oncogenes and suppressor genes [66].

The regulation of cancer cell proliferation depends on several signalling pathways and molecular mechanisms. One of the key strategies through which plant extracts can inhibit cancer cell growth is by interfering with the cell cycle, halting cancer cells at specific phases, such as G0/G1, S, or G2/M. This disruption in the normal progression of the cell cycle prevents further replication of malignant cells, eventually leading to their death [66,67,68]. Methanol extract of *Allium atroviolaceum* flowers showed antiproliferative activity against two breast cancer cell lines: MCF-7 and MDA-MB-231. MCF-7 cells were arrested in the S and G2/M phases, whereas MDA-MB-231 cells were arrested in the S phase of the cell cycle [59]. Methanolic extracts of *Andrographis nallamalayana* leaves inhibited the cell proliferation of skin cancer cell lines: A375 and B16F10. Cell cycle studies revealed that the extract acts on the G2/M phase by changing the expression of proteins involved in the G2/M phase [69]. Hexane extract of *Avicennia marina* leaves showed antiproliferative activity against HCT116 cells by arresting the G0–G1 phase of the cell cycle, while in the case of MCF-7 and HepG2 cells, the basis of this activity was the arrest of the S phase of the cell cycle [70]. Methanol extract of *Holarrhena floribunda* inhibited the proliferation of HeLa, MCF-7, and HT-29 cells by causing arrest in the G0/G1 phase of the cell cycle [61]. Another well-known compound, epigallocatechin gallate (EGCG) from green tea (*Camellia sinensis*), has been shown to arrest the cell cycle in lung cancer cells by blocking the transition from G1 to S phase. This blockage occurs through downregulation of cyclin-dependent kinases (CDKs) and inhibition of DNA synthesis [71]. EGCG’s potential as a chemopreventive agent is also supported by its ability to induce apoptosis and inhibit angiogenesis in various cancer models [72].

Cancer cells often rely on aberrant signalling pathways for growth, proliferation, and survival. Plant extracts can inhibit these pathways, particularly those involving kinases and growth factor receptors, leading to reduced tumour cell viability. The deregulation of pathways such as the PI3K/AKT/mTOR pathway, the RAS/RAF/MEK/ERK pathway, among others, leads to impaired cell proliferation and survival. Another factor leading to uncontrolled cell division is the inactivation of suppressor genes. In normal cells, the cell cycle is tightly controlled by cyclin and cyclin-dependent kinase (CDK) complexes. In cancers, excessive stimulation of CDKs is often observed through the overexpression of cyclins (e.g., cyclin D1) in breast cancer, inactivation of cell cycle blockers, such as incorrect functioning of CDK blockers (p16INK4A), lack of response to cell cycle arrest signals, e.g., by TP53 mutations [68].

Many anticancer therapies focus on inhibiting uncontrolled proliferation through kinase inhibitors (e.g., EGFR, BRAF, CDK4/6 inhibitors) or gene therapies aimed at restoring the function of suppressor genes or eliminating oncogenes. Plants are also studied as a source of compounds that may play an important role in modern anticancer therapy by inhibiting the proliferation of cancer cells.

The PI3K/AKT/mTOR pathway is frequently overactivated in cancer and is involved in cell growth, survival, and metabolism. Blocking this pathway can inhibit cancer cell proliferation and induce apoptosis. *Withania somnifera* has been shown to inhibit the PI3K/AKT pathway in breast and prostate cancer cells. By reducing AKT phosphorylation and downstream signalling through mTOR, Ashwagandha causes a significant reduction in tumour cell proliferation [73].

The MAPK/ERK pathway is another critical regulator of cell proliferation and survival, often dysregulated in cancer. A sesquiterpene lactone obtained from *Tanacetum parthenium* inhibits the growth of non-small cell lung cancer (NSCLC) cells by suppressing the B-Raf/MAPK/ERK pathway. Parthenolide suppresses non-small cell lung cancer GLC-82 cell growth via the B-Raf/MAPK/Erk pathway [74].

The Wnt/β-catenin signalling pathway plays a crucial role in regulating cell fate, and its aberrant activation is linked to the development of many cancers. Compounds from *Andrographis paniculata* have been reported to inhibit the Wnt/β-catenin signal transduction pathway, leading to reduced proliferation and induced apoptosis in colon cancer cells. This was demonstrated by decreased β-catenin levels and reduced expression of target genes such as cyclin D1 and c-Myc, which are involved in cancer cell survival [75].

In the studies by Swargiary (2021) [76], in which the antiproliferative effect of methanol extracts of four plants was tested, *Alstonia scholaris*, *Cardiospermum halicacabum*, *Hydrocotyle sibthorpioides*, and *Hypericum japonicum*, all of them showed inhibitory effects on the proliferation of the Dalton lymphoma cell line. However, the mechanism of the antiproliferative action of the tested extracts was not thoroughly explicated in these studies [76]. Ethanolic extract of dandelion (*Taraxacum officinale*) inhibited the proliferation, migration, and invasion of triple-negative breast cancer cells in the TAM microenvironment by suppressing the immunosuppressive IL-10/STAT3/PD-L1 signalling pathway [77]. Ethanolic extract of *Inula viscosa* inhibited the proliferation of the HT29 colon cancer cell line, but in this case, details of the mechanism of phytocompound action have not been investigated [78]. Polyphenolic extract from Annurca apples (*Malus domestica*) showed antiproliferative activity against MCF-7 breast cancer cells, which is probably related to the stimulation of ROS production, which can inhibit cancer cell proliferation by inducing oxidative stress, which leads to DNA damage, protein and membrane dysfunction, and activation of cell death mechanisms [79].

Unlimited cell proliferation is a hallmark of cancer and often results from mutations in oncogenes, tumour suppressor genes, and aberrant activation of key signalling pathways. Numerous studies have demonstrated that natural plant extracts exhibit strong antiproliferative activity against various cancer cell lines by targeting multiple molecular mechanisms. These include direct interference with the cell cycle, modulation of signalling pathways such as PI3K/AKT/mTOR, MAPK/ERK, and Wnt/β-catenin, as well as the regulation of cyclin-dependent kinases and tumour suppressor proteins like p53. Importantly, many plant-derived compounds selectively target cancer cells while sparing normal cells, reducing the risk of systemic toxicity. This makes them attractive candidates for use in combination with conventional therapies, such as kinase inhibitors and chemotherapeutic agents, potentially improving treatment efficacy and patient outcomes.

## 4. Anti-Angiogenic Properties of Plant-Derived Compounds

Angiogenesis is a process by which new blood vessels are formed. It is a physiological process that is involved in wound healing, tissue regeneration, the menstrual cycle, and the formation of collateral vessels to improve organ perfusion [80,81]. In tumours, there are disturbances in the regulation of angiogenesis, which leads to its stimulation. This is crucial in cancer progression, enabling the delivery of nutrients and oxygen to intensively proliferating malignant cells, which not only supports tumour development but also facilitates their migration and the formation of metastases. It can also reduce the effectiveness of chemotherapy and radiotherapy, which is possible because the blood vessels formed in pathological angiogenesis occurring in cancer are chaotically distributed, abnormally formed, and more permeable. Angiogenesis is regulated by proangiogenic and antiangiogenic molecules, and the final effect depends on their mutual ratio. They can be produced by cancer cells or can come from cells adjacent to the tumour. Common mediators and factors involved in tumour angiogenesis include vascular endothelial growth factor (VEGF) and receptors fibroblast growth factors (FGFs), angiopoietins and tie receptors (Angs), and transforming growth factor-β (TGF-β). Factors causing an increase in proangiogenic molecules include metabolic stress such as hypoxia, low pH or low glucose concentration, mechanical stress associated with pressure caused by proliferating cells, immune response, or genetic mutations such as activation of oncogenes, deletion of tumour suppressor genes controlling the production of angiogenesis regulators [81,82,83,84].

The attempt to inhibit angiogenesis is a promising approach supporting cancer therapy by inhibiting tumour progression. Pathological angiogenesis inhibitors combined with chemotherapeutics or immunotherapy may be an effective solution in cancer treatment. The use of angiogenic inhibitors such as protease inhibitors, endothelial cell migration and proliferation inhibitors, angiogenic growth factor inhibitors, endothelial cell surface matrix protein inhibitors, or inhibitors with unique mechanisms may contribute to reducing cancer mortality [80,85].

Plants are excellent biochemists, capable of synthesising molecular structures that serve as valuable scaffolds for the development of new, safe, and effective drugs. Accordingly, researchers are actively seeking new plant species whose phytochemicals exhibit antiangiogenic properties. Notably, several plant-derived compounds have been identified as potential allies in this anticancer mechanism—the inhibition of new blood vessel formation within tumour tissue [86].

A universal method for this type of experimentation is the in ovo CAM test—a biological model that utilises the chorioallantoic membrane of the chicken embryo to study angiogenesis, tumour development, toxicity, biocompatibility, and the efficacy of drugs and bioactive substances. This model is based on the use of a live avian embryo (typically *Gallus gallus domesticus*) developing inside an incubated egg (in ovo), with the CAM membrane becoming easily accessible and highly vascularised from around day 7 of incubation, making it suitable for experimental manipulation. After application of the tested material, morphological and physiological changes in the CAM are monitored, such as the induction or inhibition of angiogenesis, invasion of tumour cells into the membrane, as well as toxic effects and inflammatory or immunological responses. Thus, the CAM test represents a versatile, ethically acceptable research tool, combining low cost, ease of implementation, and the biological complexity of the test environment. Thanks to the possibility of conducting experiments in ovo, it serves as an important intermediate step between in vitro studies and tests on higher animals [87].

In ovo test of aqueous extract of *Melissa oficinalis* leaves showed its angiogenesis-inhibiting effect even at low concentrations with simultaneous lack of toxicity [88]. As well, ethanolic extracts of Greek *Olea europaea* leaves showed antiangiogenic activity in the CAM test [89]. Ethanolic extracts of *Bidens tripartite* herb, *Galium verum* herb, and *Rumex hydrolapathum* root showed the ability to modify angiogenesis by affecting the level of angiogenic/angiostatic mediators: PDGF (platelet-derived growth factor), HGF (hepatocyte growth factor), IL-8 (interleukin-8/CXCL8), IL-6 (interleukin-6), TIMP-1 (tissue inhibitor of metalloproteinases-1) and MMP-9 (matrix metalloproteinase-9). The herbal extract of *Bidens tripartita* exhibited the most potent antiangiogenic activity [90]. In the studies of Kei and Raju (2022) [91], the antiangiogenic activity of the methanolic and aqueous extract of *Moringa oleifera* leaves was tested using the in ovo CAM method. It showed that aqueous extract of *Moringa oleifera* leaves significantly reduced the number of blood vessels and showed stronger antiangiogenic properties than sunitinib (a standard targeted drug that, by inhibiting tyrosine kinases, blocks tumour growth and its vascularisation) and methanol [91]. The ethanolic extract of *Andrographis echioides* exhibited a concentration-dependent antiangiogenic effect in the chorioallantoic membrane of chickens, with activity increasing proportionally with the extract concentration [92]. Aqueous extract of *Agrostemma githago* seeds showed the ability to inhibit angiogenesis, which was associated with changes in the release of VEGF, MMP2/9, and ANG2 (angiopoietin-2; crucial in vascular remodelling and tumour angiogenesis) from cancer and endothelial cell lines [93]. As well, aqueous and ethanolic extracts of *Nelumbo nucifera* showed an inhibitory effect on VEGF-induced angiogenesis: inhibition of VEGF-induced tube formation and CAM angiogenesis in vivo was observed [94]. Similarly, ethanolic and aqueous extracts of *Withania somnifera* exhibited antiangiogenic activity associated with downregulation of various angiogenic growth factors [95]; methanolic extracts of *Alternanthera sessilis*, *Alstonia scholaris*, and *Anogeissus acuminata* showed antiangiogenic properties in studies using the in ovo CAM method [96]. In contrast, the ethanolic extract of *Melilotus indicus* showed antiangiogenic activity in in vivo studies. In vivo studies, including measurements of tumour mass and histopathological tests, showed an antiangiogenic effect of n-hexane extract of *Annona reticulata* seeds, and microarray analysis showed reduced expression of major tumour proangiogenic proteins [97]. In vitro and in vivo studies showed that aqueous extracts of *Euphorbia pekinensis* have antiangiogenic properties by inhibiting the expression of most genes related to cancer-related angiogenesis. In addition, the study showed that the plant extracts were able to inhibit angiogenesis by suppressing blood vascularisation in the in vivo CAM test [98]. Methanolic extracts of *Cassia occidentalis*, *Callistemon viminalis*, *Cleome viscosa*, and *Mimosa hamata* showed antiangiogenic activity. In addition, by suppressing blood vascularisation in the in vivo CAM test [99].

The cited studies have demonstrated that numerous plant extracts (aqueous, ethanolic, methanolic, and n-hexane) possess antiangiogenic potential, primarily evidenced using the in ovo CAM (chorioallantoic membrane) assay. Plants such as *Melissa officinalis*, *Moringa oleifera*, *Bidens tripartita*, *Andrographis echioides*, *Agrostemma githago*, *Nelumbo nucifera*, and *Withania somnifera* have shown the ability to inhibit angiogenesis, often through the regulation of key mediators such as VEGF, ANG2, MMP-2/9, PDGF, HGF, IL-6, IL-8, and TIMP-1. Some studies have been extended to in vivo and in vitro models, confirming both the mechanisms of action and the effects of these extracts on the expression of angiogenesis-related genes. Further research is needed to elucidate the precise signalling pathways modulated by specific extracts (e.g., MAPK/ERK, PI3K/AKT, JAK/STAT, VEGF/VEGFR, Tie2/ANG2).

Comparative studies with conventional antiangiogenic drugs (e.g., sunitinib, bevacizumab) are also recommended to evaluate the potential of plant extracts as adjunctive or alternative therapies. Notably, combining plant-based compounds with anticancer drugs may represent a promising strategy to enhance therapeutic efficacy while minimising adverse effects.

## 5. Immunomodulatory Potential of Plant Extracts in Cancer Therapy

Cancer patients mount an immune response against the tumour, but ultimately, the tumour cells successfully evade destruction. Although lymphocytes and NK (“natural killer”; responsible for the immunological control of tumours, they can recognise and eliminate cancer cells that evade detection by T lymphocytes) cells try to fight them, the tumour uses mechanisms that suppress the immune system. One such mechanism is the production by tumour cells of immune-suppressing substances such as cytokines and prostaglandins, which promote a Th2 response. Dominance of the Th2 immune response may suppress the more cytotoxic, antitumor Th1 response, potentially promoting tumour progression. This results in a weakened antitumor response by reducing the level of interleukin-2 and inhibiting the proliferation and functioning of NK, T helper, and cytotoxic T cells. Another mechanism is that immune-resistant variants of tumour cells arise because of MHC (major histocompatibility complex) I and II selection and mutations in antigen processing, which reduce their antigenicity (they reduce MHC expression and disrupt antigen presentation). In addition, tumour cells can destroy T lymphocytes, causing their death or attacking them through the expression of Fas ligand (FasL). Under physiological conditions, Fas ligand is primarily expressed on cytotoxic T lymphocytes (CD8^+^) and natural killer (NK) cells, where it functions to eliminate target cells, such as infected or cancerous cells. FasL binds to the Fas receptor (CD95) on these target cells and induces their apoptosis. Tumours that overexpress FasL on their surface disrupt the normal functioning of the immune system. Upon contact with such tumours, T lymphocytes expressing Fas receptors (CD95) undergo apoptosis themselves. This phenomenon is called “tumour-induced immunosuppression.”

In recent years, immunotherapy has become increasingly popular in cancer research and clinical oncology. Immune-based cancer therapies and chemotherapeutic drugs can not only stimulate an immune response against the tumour, but also affect its microenvironment, which significantly changes the approach to cancer treatment [100].

The immunomodulatory effects of targeted anticancer drugs can result from both their effects on cancer cells and from their effects on immune system cells, modifying their functions. Both mechanisms of immunomodulation in anticancer therapy can act directly, enhancing a specific effect, or indirectly, weakening the antagonistic effect. For example, specialised anticancer drugs can support the immune system by stimulating the secretion of proinflammatory cytokines or inhibiting the action of substances that suppress the immune response. Targeted anticancer therapy can modulate the immune response, although this is not always associated with the elimination of cancer or immune cells. It can induce a highly immunogenic type of cancer cell death, starting a cycle of interactions between the tumour and the immune system, or selectively remove immunosuppressive cells, such as regulatory T cells (TREG—they are a specialised subset of CD4^+^ T lymphocytes involved in maintaining immune tolerance and preventing autoimmune responses by suppressing excessive immune activation), which promote the development of the disease [101].

Despite the continuous development of immunotherapy, the discovery of natural substances that modulate immunity and stimulate innate immune cells also opens new possibilities in cancer treatment. Plants are a rich source of immunomodulatory substances that exhibit promising anticancer activity while not causing many side effects [102].

Tested ethanolic extracts of *Tinospora cordifolia*, *Boerhaavia diffusa*, *Berberis aristata*, and *Ocimum basilicum* showed immunomodulatory properties. An increase in interferon gamma production was observed in the presence of *Tinospora cordifolia* and *Ocimum basilicum* extracts, indicating that these extracts may support the immune response and improve the body’s natural ability to fight cancer [103]. Ethanolic extract of *Populus nigra* exerted immunomodulatory effects on human primary dendritic cells and worked effectively even at low concentrations [104]. Aqueous extract of *Hordeum vulgare* provoked immunomodulatory activity by improving the ability of NK cells to recognise and eliminate human colon cancer cells without any side effects on normal colon epithelial cells [105]. Alcoholic extract from the roots of Ashwagandha has an immunomodulatory effect that promotes immunogenic death of T-cell leukaemia. In studies conducted on mice using models of myelosuppression induced by cyclophosphamide, azathioprine, or prednisolone, this extract was observed to increase the number of blood cells, improve bone marrow cellularity, and increase the number of cells expressing α-esterase [106]. *Astragalus membranaceus* reduces immunosuppression by stimulating the activity of M1 macrophages and supporting the ability of T cells to destroy cancer cells in the tumour microenvironment (TME). AM has also been shown to enhance systemic immunity, which may contribute to increased efficacy of chemotherapy and reduced risk of metastasis [107]. In the study conducted by Kim et al. [108], the immunomodulatory effect of commercially available *Echinacea purpurea* extract was evaluated in a mouse model with cyclophosphamide-induced immunosuppression. It was found that the extract contributed to the improvement of parameters related to the spleen and thymus. Additionally, increased proliferation of splenocytes, higher NK cell activity, as well as an increase in the number of T cells and cytokine levels were observed in mice exposed to cyclophosphamide.

Cancer cells have developed sophisticated mechanisms to evade immune surveillance, despite the initial anti-tumour response mounted by lymphocytes and NK cells. Because of it, in recent years, immunotherapy has gained prominence in oncology. Targeted anticancer agents not only affect tumour cells but can also modulate immune responses. Plant-derived compounds offer a promising complementary approach in therapy due to their dual action: anticancer and immunomodulatory, often with fewer side effects than conventional therapies.

## 6. Mitigation of Chemotherapy-Induced Side Effects Using Plant Extracts

The side effects of chemotherapy continue to be a major problem in cancer therapy, raising concerns among patients and significantly reducing their quality of life. Despite the rapid development of medicine and the existence of drugs aimed at eliminating the troublesome effects of chemotherapy-based therapy, they are not always effective, do not show protective effects against the long-term effects of chemotherapy, and their use may also be burdened with additional side effects. The most common side effects of chemotherapy include nausea and vomiting and inflammation of the oral mucosa and the gastrointestinal tract, leading to ulceration. This may result in anorexia, poor absorption, anaemia, weight loss, fatigue, and an increased risk of sepsis [109]. Other side effects are related to the cardiotoxicity of chemotherapy drugs, which may lead to changes in blood pressure, electrocardiographic changes, arrhythmias, pericarditis, myocarditis, cardiomyopathy, heart attack, heart failure, or thrombosis [110]. In turn, nephrotoxicity of conventional anticancer drugs leads to acute kidney injury (via tubular damage, tubulointerstitial nephritis, glomerular disease, and thrombotic microangiopathy), long-term loss of renal function, and electrolyte disturbances [111]. Neurotoxicity of chemotherapy affects the composition of cerebrospinal fluid, which is associated with cognitive impairment [109]. Loss of homeostatic control of reactive oxygen species, inflammation, DNA damage, and mutagenicity are the main mechanisms responsible for the side effects of chemotherapy that reduce the quality of life of patients undergoing treatment. Reactive oxygen species (ROS) play an important role in many cell-signalling pathways essential for the proper functioning of the organism. However, their excess can lead to serious oxidative damage by interacting with proteins, lipids, and DNA, resulting in adverse consequences for the host. Many anticancer drugs and radiotherapy lead to increased production of ROS, depending on the applied dose and time of exposure. Long-term elevation of free radicals can exceed the protective capacity of cellular antioxidant systems, resulting in serious adverse effects. As a result of cancer therapy, depletion of antioxidant resources is often observed because they are intensively used by chemotherapeutic agents. This results in a decreased ability to neutralise free radicals, accumulation of ROS, and an increase in the level of malondialdehyde (MDA)—a product of lipid peroxidation—in plasma. Healthy tissue cells use antioxidants and ROS-producing enzymes to maintain redox balance, but chemotherapy and radiation can disrupt their function, leading to an uncontrolled increase in ROS due to direct effects on antioxidant enzymes. Additionally, these drugs can pathologically activate ROS-producing enzymes. Among them, NADPH oxidases (NOX) play a dominant role, especially the isoforms NOX-1, NOX-2, and NOX-4, which are involved in cisplatin-induced ROS generation, leading to hearing and kidney damage. It is known that oxidative stress can impair mitochondrial function and activate proapoptotic pathways, especially those related to MAPK kinases. Studies have shown that activation of JNK and p38 MAPK kinases is associated with p53- and Bcl-2-dependent apoptosis in response to oxidative stress induced by anticancer therapy. Therefore, in oncological therapy, it is worth considering approaches that limit oxidative stress or block proapoptotic pathways to improve the condition of patients.

Due to the side effects occurring during chemotherapy, it is important to develop measures to prevent and limit both short-term and long-term adverse effects of popular chemotherapy drugs to improve the quality of life of patients [109].

As it has been mentioned above, natural bioactive compounds of plant origin may contribute to reducing the side effects of chemotherapy [109]. Aqueous extract prepared from eight herbs used in traditional Chinese medicine (*Astragalus membranaceus*, *Glycyrrhiza uralenisi*, *Codonopsis pilosula*, *Angelica sinensis*, *Citus reticulate*, *Cimicifuga heracleifolia*, *Bupleurum chinense*, and *Atractylodes macrocephala*) has an inhibitory effect on kidney damage caused by 5-flurouracil, which is probably related to the reduction in apoptosis and necrosis of renal tubular epithelial cells, thanks to its antioxidant properties. Hydroalcoholic extract of *Rubia cordifolia* showed an inhibitory effect on kidney damage caused by cisplatin, which may be due to the strong antioxidant properties of the extract [112]. Ethanolic extract of *Morus nigra* leaves showed a protective effect on liver tissues exposed to methotrexate, which was probably due to the antioxidant and cytoprotective effects of the extract [113]. Aqueous extract of *Morinda citrifolia* fruit demonstrated activity in alleviating methotrexate-related toxicity [114]. *Ceiba pentandra* extract showed protection activities in the kidneys against methotrexate-induced damage due to its antioxidant, antiapoptotic, and anti-inflammatory properties [115]. *Ginkgo bioloba* extract has a protective effect on liver tissues exposed to methotrexate, reducing the expression of TNF-α, p-JNK, caspase-3, and COX-2 pathways, inhibiting ROS production, increasing hepatic GSH and GST levels, and reducing NO levels [116].

Reducing oxidative stress and apoptosis in non-tumour tissues is a promising strategy to mitigate chemotherapy side effects. Natural plant-derived compounds with antioxidant, anti-inflammatory, and antiapoptotic properties have shown protective effects against drug-induced toxicity in preclinical studies. Herbal extracts from plants such as *Morus nigra*, *Rubia cordifolia*, *Ginkgo biloba*, and *Morinda citrifolia* may attenuate tissue damage caused by common chemotherapeutics (e.g., cisplatin, methotrexate, 5-Fluorouracil), primarily through antioxidant action and modulation of apoptotic signalling. It seems that integrating plant-based antioxidants with conventional chemotherapy may offer a dual benefit—maintaining antitumor efficacy while protecting healthy tissues. However, clinical validation is necessary to confirm efficacy, optimal dosing, and safety of these adjunctive therapies.

## 7. Challenges and Future Prospects for the Use of Plant Extracts in Cancer Treatment

As can be seen from the presented review, plant extracts have shown significant potential in cancer therapy due to their ability to target multiple signalling pathways involved in tumour growth and progression. Many of these natural compounds possess anti-inflammatory, antioxidant, proapoptotic, and antiproliferative properties, what has been summarised in Table 1. It seems that they can be used as complementary agents alongside conventional chemotherapy to enhance therapeutic efficacy, reduce drug resistance, and minimise side effects.

Despite great hopes placed in the use of phytochemicals in anticancer therapies, this approach encounters challenges from modern medicine and pharmaceutical principles. It is related to the variable composition of plant extracts, which depends on environmental and climate changes (seasons, stresses, place of growth, e.g., altitude), the different extraction efficiency, poor solubility of isolated compounds, and their poor stability, which contributes to limiting their bioavailability [117]. Although the use of nanotechnology, e.g., nanoformulations, can improve these properties [118], it is difficult to change the pharmaceutical approach to a drug as a substance with it is difficult to change the pharmaceutical approach to a drug as a substance with a precisely defined composition and dose.

The creation of anticancer drugs based on substances from natural sources is a multi-stage process that begins in the laboratory and ends with the implementation of effective therapies for patients. This process includes preclinical and clinical phases, each of which plays a key role in the transformation of plant raw materials into safe and effective therapeutic agents.

The preclinical phase includes in vitro studies and experiments on animal models, which allow for the assessment of the action, safety, and pharmacological properties of potential drugs. Only after a positive assessment of these parameters do the substances move on to clinical trials, in which they are tested on humans to determine their actual therapeutic usefulness and possible side effects [119]. Moreover, relying predominantly on in vitro and in ovo models in research significantly limits their translational relevance. Traditional 2D cell cultures fail to accurately replicate the complex architecture of tissues, intercellular interactions, extracellular matrix components, and the full spectrum of pharmacokinetic and pharmacodynamic processes occurring in living organisms. As a result, many promising findings from such studies do not translate successfully into animal models or clinical trials. Although three-dimensional models, such as organoids and spheroids, better mimic tissue structure and can sometimes predict toxicity, they still fall short of capturing the complete dynamics of drug distribution, metabolism, and systemic interactions between organs. In ovo models, such as the CAM assay, allow for the observation of processes like angiogenesis and tumour growth. However, they come with considerable limitations, including the absence of a developed immune system, a narrow observation window, difficulties in drug administration, lack of fetal-maternal exchange and metabolism, and significant interspecies differences, which reduce their translational applicability. Therefore, to thoroughly evaluate the therapeutic potential and safety profile of a given substance, the use of in vivo models is essential. Only these models provide insights into the complex responses of the entire organism—including immune reactions, metabolism, biodistribution, and potential side effects—which are crucial for advancing therapeutic development [120,121,122].

Clinical trials, a key stage in drug development, are divided into four phases. Phase I aims to assess safety and determine appropriate doses, usually involving a small group of healthy volunteers or patients. In Phase II, the drug is tested on a larger number of patients to assess efficacy and further monitor safety. Phase III, conducted on a large population, compares the new drug with current treatment standards, providing data necessary for product registration. Phase IV involves post-marketing surveillance, focusing on long-term effects and safety [119].

An example of a natural compound with potential anticancer activity is artemisinin, derived from the plant *Artemisia annua*. Although primarily known for its antimalarial activity, preliminary studies have shown that it also has activity against various types of cancer cells, including leukaemia, colon, breast, lung, and pancreatic cancer. In clinical trials, artemisinin has gone through successive phases, starting with the assessment of safety and tolerability (Phase I), through the analysis of therapeutic efficacy (Phase II), and finally, comparison with conventional therapies (Phase III). One randomised, double-blind study in patients with colon cancer showed good tolerability and promising anticancer activity of this substance [119,123].

Curcumin, an active ingredient in turmeric, has also been studied for its anticancer potential. Many clinical trials have assessed its effect on various types of cancer. Phase I/II studies have shown that it is well tolerated and safe. In patients with metastatic colorectal cancer, curcumin combined with chemotherapy has shown positive therapeutic effects. Other studies have assessed its effects on cancer biomarkers, anti-inflammatory effects, and its efficacy as a supportive treatment for breast, pancreatic, cervical, endometrial, prostate, and multiple myeloma cancers. Curcumin has also been studied as a potential radiosensitiser and radioprotector, which further expands the possibilities of its therapeutic use [119,124,125].

An example of the limitations associated with the use of curcumin is its very low bioavailability. This compound is poorly soluble in water, undergoes rapid hepatic metabolism, and has a short plasma half-life, which significantly limits its effectiveness in clinical settings [126,127].

Genistein, a soy isoflavonoid, has been the subject of Phase I and II studies in the treatment of prostate cancer. Among other things, its pharmacokinetics, toxicity, and ability to regulate the expression of genes associated with cancer metastasis were assessed. Clinical studies have shown that genistein is well tolerated even at high doses, and its use was associated with a significant reduction in PSA levels and the expression of genes promoting tumour invasion, such as MMP-2. These results indicate that genistein can modulate pathways responsible for cancer progression and exhibit anticancer activity in humans [128].

The extract that has undergone clinical trials is P2Et—an extract from *Caesalpinia spinosa*. No serious adverse effects were observed in healthy volunteers taking up to 600 mg daily, confirming its safety and potential for further clinical trials. The standardised P2Et extract obtained from *Caesalpinia spinosa* has shown antioxidant and direct antitumor activity, but also activation of specific immune response through the induction of tumour immunogenic cell death in breast and melanoma cancer models [129,130].

All these examples show that natural substances can be a valuable source of new anticancer drugs. However, their development requires rigorous testing at every stage—from preclinical to clinical—to ensure the safety and efficacy of the therapy. Hence, most of them are isolated ingredients and not complex extracts.

On the other hand, it is well known that fractionation, identification, and isolation of bioactive compounds contained in plant extracts are time-consuming and expensive procedures. At the same time, several publications [131,132,133] confirm that the use of whole/complexed plant extracts in anticancer therapy is often considered more beneficial than the use of single, isolated plant compounds due to several important aspects.

First of all, complex plant extracts contain many different bioactive components that can act synergistically, i.e., mutually reinforcing their anticancer effects. Unlike a single compound, which usually has a limited effect directed at one mechanism, a complex of compounds present in the extract can affect cancer cells at different levels, for example by inhibiting their proliferation, inducing apoptosis, or blocking the development of blood vessels necessary for tumour growth. Thanks to this, the action of the extract is more versatile and effective. In addition, the use of whole extracts reduces the risk of cancer cells developing resistance to therapy. Cancers can often quickly become resistant to the action of a single substance, while the multi-component action of plant extracts makes it difficult for them to adapt to treatment.

Moreover, extracts also contain different compounds that can reduce the toxicity of the main active ingredients, which reduces the risk of side effects and improves the tolerance of therapy. In addition, complex plant extracts often contain substances that facilitate the absorption and metabolism of active ingredients, which increases their bioavailability and effectiveness.

It is known that bioactive secondary metabolites in plants constitute a highly effective survival strategy in harmful environmental conditions, defence against pathogens, etc.; thus, evolution has certainly preserved and promoted the most favourable (optimal) compositions of phytochemicals in plant vacuoles. Perhaps, also for this reason, they should not be reduced and suppressed by fractionating and isolating individual components?

Finally, the isolation of individual compounds often requires complex chemical processes that can lead to the loss or change of the properties of other important substances present in the plant. Therefore, maintaining a full phytochemical profile in the extract allows the use of the natural balance of ingredients, which promotes more effective and safer anticancer treatment.

For these reasons, therapy based on whole plant extracts may be more effective than that based only on single isolated compounds.

Although all the above-mentioned reasons suggest that therapy based on complex plant extracts may be more effective than therapy based solely on single isolated compounds, it should be remembered that some elements of plant extracts may unfortunately exhibit toxicity to healthy cells or cause adverse effects such as myelosuppression or organ damage. Therefore, thorough toxicological studies of extracts are absolutely necessary before their clinical use [134,135].

To overcome all described challenges, it is advisable to expand the rather limited research in the field of herbal medicine to provide sufficient information on their efficacy, mechanisms of action, and safety. Standardisation of complex plant extracts seems to be the most difficult challenge but solving this problem is extremely important in the context of preventive actions and potential benefits for patients worldwide.

## Figures and Tables

**Table 1:** Overview of antitumor activities of the discussed plant extracts.

Latin Name	English Name	Anticancer Activity	References
*Agrostemma githago*	Corncockle	Antiangiogenic: angiogenesis inhibition—associated with changes in the release of VEGF, MMP2/9, and ANG2 from cancer and endothelial cell lines	[93]
*Allium atroviolaceum*	Dark Purple Garlic	Proapoptotic: apoptosis-inducing effect in MCF-7 and MDA-MB-231 by extrinsic and intrinsic pathways—caspase- and Bcl-2-dependent and p53 transcription-independent Antiproliferative: inhibition of MCF7 (arrest in the S and G2/M phases) and MDA-MB-231 (arrest in the S phase) cell proliferation	[59]
*Alstonia scholaris*	Maidenhair Tree	Antiproliferative: inhibition of Dalton lymphoma cell proliferation Antiangiogenic: angiogenesis inhibition	[76,96]
*Alternanthera sessilis*	Sessile Joyweed	Antiangiogenic: angiogenesis inhibition	[96]
*Andrographis echioides*	False Waterwillow	Antiangiogenic: angiogenesis inhibition	[92]
*Andrographis paniculata*	Green Chiretta	Antiproliferative, proapoptotic: inhibition the Wnt/β-catenin signal transduction pathway, leading to reduced proliferation and induced apoptosis in colon cancer cells	[75]
*Andrographis nallamalavana*	-	Antiproliferative: inhibition of A 375 and B16F10 cell proliferation—acts on the G2/M phase by changing the expression of proteins involved in the G2/M phase	[69]
*Annona cherimola*	Cherimoya	Proapoptotic: apoptosis-inducing effect in MDA-MB-231—release of cytochrome C, an increase in p21 levels, and an increase in the Bax/Bcl-2 ratio, and acute myeloid leukaemia lines—PARP cleavage, activation of caspases 8 and 9, and an increase in the Bax/Bcl-2 ratio	[56,57]
*Annona reticulata*	Custard Apple	Antiangiogenic: angiogenesis inhibition—reduced expression of major proangiogenic proteins	[97]
*Anogeissus acuminata*	-	Antiangiogenic: angiogenesis inhibition	[96]
*Astragalus membranaceus*	Astragalus Root	Immunomodulatory: reduces immunosuppression by stimulating the activity of M1 macrophages and supporting the ability of T cells to destroy cancer cells in the tumour microenvironment	[107]
*Avicennia marina*	Grey Mangrove	Proapoptotic: apoptosis-inducing effect in MDA-MB-231—DNA fragmentation, increased p53 expression, and decreased Bcl2 protein levels Antiproliferative: inhibition of HCT116 (arrest the G0-G1 phase), MCF-7, and HepG2 (arrest of the S phase) cells	[60,70]
*Berberis aristata*	Indian Barberry	Immunomodulatory effect	[103]
*Bidens tripartite*	Three-lobe Beggarticks	Antiangiogenic: angiogenesis inhibition—affecting the level of angiogenic/angiostatic mediators: PDGF, HGF, IL-8, IL-6, TIMP-1, and MMP-9	[90]
*Boerhaavia diffusa*	Punarnava/Spreading Hogweed	Immunomodulatory effect	[103]
*Callisetmon viminalis*	Weeping Bottlebrush	Antiangiogenic: angiogenesis inhibition	[99]
*Camellia sinensis*	Green Tea	Antiproliferative: arrest the cell cycle in lung cancer cells by blocking the transition from G1 to S phase	[71]
*Cardiospermum halicacabum*	Balloon Vine	Antiproliferative: inhibition of Dalton lymphoma cell proliferation	[76]
*Cassia occidentalis*	Coffee Senna	Antiangiogenic: angiogenesis inhibition	[99]
*Ceiba pentandra*	Kapok Tree	Reducing the side effects: protection activities the kidneys against methotrexate-induced damage	[115]
*Ceratonia siliqua*	Carob Tree	Proapoptotic: apoptosis-inducing in colon cancer cells	[50]
*Cleome viscosa*	Asian Spiderflower	Antiangiogenic: angiogenesis inhibition	[99]
*Cucurbita ficifolia*	Fig-leaved Gourd	Proapoptotic: apoptosis-inducing effect in MCF-7 breast cancer cells—modification of the expression levels of caspase-3, caspase-8, and caspase-9 genes and p53, FAS, FADD, Bax, and Bak	[51]
*Curcuma longa*	Turmeric	Proapoptotic: various signalling molecules involved in apoptosis, including p53, Bax, Bcl-2, and caspase-3, promoting cancer cell death (breast and prostate cancer cell lines) while sparing normal cells	[53]
*Echinacea purpurea*	Purple Coneflower	Immunomodulatory: improvement of parameters related to the spleen and thymus, increased proliferation of splenocytes, higher NK cell activity, increase in the number of T cells and cytokine levels	[108]
*Elaeaganus angustifolia*	Russian Olive	Proapoptotic: apoptosis-inducing effect in MDA-MB-231 and MDA-MB-436 by affecting the expression of proapoptotic and antiapoptotic genes	[55]
*Euphorbia pekinensis*	Peking Spurge	Antiangiogenic: angiogenesis inhibition—inhibiting the expression of most genes related to cancer-related angiogenesis	[98]
*Ficus exasperata*	Sandpaper Fig	Proapoptotic: apoptosis-inducing in PC-3 cells via the Bax/Cytochrome C/Caspase 3-9 signalling pathway	[54]
*Galium verum*	Lady’s Bedstraw	Antiangiogenic: angiogenesis inhibition—affecting the level of angiogenic/angiostatic mediators: PDGF, HGF, IL-8, IL-6, TIMP-1, and MMP-9	[90]
*Ginkgo biloba*	Maidenhair Tree	Reducing the side effects: protective effect on liver tissues exposed to methotrexate	[116]
*Holarrhena floribunda*	False Rubber Tree	Proapoptotic: apoptosis-inducing effect in HeLa, MCF-7, and HT-29 Antiproliferative: inhibition of HeLa, MCF-7, and HT29 cell proliferation—acts on the G2/M phase by changing the expression of proteins involved in the G2/M phase—arrest in the G0/G1 phase of the cell cycle.	[61]
*Hordeum vulgare*	Barley	Immunomodulatory effect: improving the ability of NK cells to recognise and eliminate human colon cancer cells	[105]
*Hydrocotyle sibthorpioides*	Lawn Marshpennywort	Antiproliferative: inhibition of Dalton lymphoma cell proliferation	[76]
*Hypericum japonicum*	Japanese St. John’s Wort	Antiproliferative: inhibition of Dalton lymphoma cell proliferation	[76]
*Innula viscosa*	Sticky Inula	Antiproliferative: inhibition of HT29 cell proliferation	[78]
*Ipomoea carnea*	Bush Morning Glory	Proapoptotic: apoptosis-inducing effect in breast cancer cells	[58]
*Malus domestica*	Annurca Apples	Antiproliferative: inhibition of MCF-7 cell proliferation	[79]
*Melissa oficinalis*	Lemon Balm	Antiangiogenic: angiogenesis inhibition	[88]
*Mimosa hamata*	-	Antiangiogenic: angiogenesis inhibition	[99]
*Morinda citrifolia*	Noni/Indian Mulberry	Reducing the side effects: activity in alleviating methotrexate-related toxicity	[114]
*Moringa oleifera*	Drumstick Tree/Moringa	Antiangiogenic: angiogenesis inhibition	[91]
*Morus nigra*	Black Mulberry	Reducing the side effects: protective effect on liver tissues exposed to methotrexate	[113]
*Nelumbo nucifera*	Sacred Lotus	Antiangiogenic: angiogenesis inhibition	[94]
*Ocimum basilicum*	Sweet Basil	Immunomodulatory effect: increase in interferon gamma production	[103]
*Olea europaea*	Olive Tree	Antiangiogenic: angiogenesis inhibition	[89]
*Phyllanthus muellerianus*	Müller’s Leaf-flower	Proapoptotic: apoptosis-inducing in PC-3 cells via the Bax/Cytochrome C/Caspase 3-9 signalling pathway	[54]
*Phoenix dactylifera*	Date Palm	Proapoptotic: apoptosis-inducing effect in MDA-MB-231	[52]
*Populus nigra*	Black Poplar	Immunomodulatory effect: human primary dendritic cells	[104]
*Rubia cordifolia*	Indian Madder	Reducing the side effects: inhibitory effect on kidney damage caused by cisplatin	[112]
*Rumex hydrolapathum*	Water Dock	Antiangiogenic: angiogenesis inhibition—affecting the level of angiogenic/angiostatic mediators: PDGF, HGF, IL-8, IL-6, TIMP-1, and MMP-9	[90]
*Tanacetum parthenium*	Feverfew	Antiproliferative: inhibition of the growth of non-small cell lung cancer (NSCLC) cells by suppressing the B-Raf/MAPK/ERK pathway	[74]
*Taraxacum officinale*	Common Dandelion	Antiproliferative: inhibition of triple-negative breast cancer cell proliferation—in the TAM microenvironment by suppressing the immunosuppressive IL-10/STAT3/PD-L1 signalling pathway	[77]
*Tinospora cordifolia*	Heart-leaved Moonseed/Guduchi	Immunomodulatory effect: increase in interferon gamma production	[103]
*Withania somnifera*	Ashwagandha/Indian Ginseng	Proapoptotic: apoptosis induction by activating both the intrinsic and extrinsic apoptotic pathways Antiproliferative: reduction in AKT phosphorylation and downstream signalling through mTOR Antiangiogenic: angiogenesis inhibition—associated with downregulation of various angiogenic growth factors Immunomodulatory: promotion of immunogenic death of T-cell leukaemia	[62,73,95,106]

## Abbreviations

The following abbreviations are used in this manuscript:

*RAS*	rat sarcoma viral oncogene homolog
*MYC*	avian myelocytomatosis oncogene homolog
*TP53*	gene encoding the p53 tumour protein
*RB1*	gene encoding pRb retinoblastoma protein
pRb	retinoblastoma protein; controls G1 to S phase transition during cell cycle
Bcl-2	B-cell Lymphoma 2; family of proteins that regulate cell apoptosis
Bak	Bcl-2 homologous antagonist/killer; apoptotic protein
Bax	Bcl-2-associated X protein; apoptotic protein
Bcl-xL	B-cell lymphoma extra-large; antiapoptotic protein
MMPs	extracellular matrix metalloproteinases
*BRCA1*, *BRCA2*	breast cancer genes 1 and 2; tumour suppressor genes involved in DNA repair through homologous recombination
*MLH1*	MutL Homolog 1; the human homolog of the bacterial MutL gene key in DNA mismatch repair pathway
TNF	tumour necrosis factor
DISC	death-inducing signalling complex
FasL	Fas ligand; binds to the Fas receptor to initiate apoptosis
TNF-α	tumour necrosis factor alpha; proinflammatory cytokine
MOMP	mitochondrial outer membrane permeabilisation
IAP	inhibitor of apoptosis proteins
NF-κB	nuclear factor kappa-light-chain-enhancer of activated B cells; a family of transcription factor proteins
BIR	baculoviral IAP repeat domains
Mcl-1	antiapoptotic protein
MCF-7	Michigan cancer foundation-7; human *breast* cancer cell line
p53	tumour suppressor protein responsible for monitoring and controlling the genetic integrity of cells
FAS	cell surface death receptor that upon binding FasL triggers apoptosis via DISC formation
FADD	FAS-associated death domain; apoptosis signal mediator
MDA-MB-231	MD Anderson—metastatic breast cancer line 231; triple-negative human breast cancer cell line
MDA-MB-436	MD Anderson—metastatic breast cancer line 436; triple-negative human breast cancer cell line
MCF-7	human breast adenocarcinoma cell line; oestrogen receptor-positive
MCF 10A	non-cancerous human mammary epithelial cell line
p21	cyclin-dependent kinase inhibitor 1A; cell cycle suppressor protein
PARP	poly(ADP-ribose)-polymerase
HeLa	epithelial cervical cancer cell line derived from Henrietta Lacks
HT-29	human colorectal adenocarcinoma cells
HepG2	human hepatocellular carcinoma cells
EGCG	epigallocatechin gallate
CDKs	cyclin-dependent kinases
EGFR	epidermal growth factor receptor; kinase inhibitor
BRAF	B-rapidly accelerated fibrosarcoma (B-type Raf kinase) kinase
CDK4/6	cyclin-dependent kinases 4 and 6; inhibitors
VEGF	vascular endothelial growth factor
FGFs	receptors fibroblast growth factors
Angs	angiopoietins and tie receptors
TGF-β	transforming growth factor-β
CAM test	a biological model that utilises the chorioallantoic membrane of the chicken embryo to study angiogenesis, tumour development, toxicity
PDGF	platelet-derived growth factor
HGF	hepatocyte growth factor
IL-8	interleukin-8/CXCL8
IL-6	interleukin-6
TIMP-1	tissue inhibitor of metalloproteinases-1
MMP-9	matrix metalloproteinase-9
ANG2	angiopoietin-2; crucial in vascular remodelling and tumour angiogenesis
NK	“natural killer”; responsible for the immunological control of tumours and virus-infected cells
MHC	major histocompatibility complex
FasL	Fas ligand
Fas	receptor CD95
TREG	regulatory T cells; suppress immune responses, often elevated in tumour microenvironments
TME	tumour microenvironment
ROS	reactive oxygen species
NOX	NADPH oxidase
JNK	c-Jun N-terminal kinase
p38	p38 mitogen-activated protein kinase
GSH	reduced glutathione
GST	glutathione s-transferase
COX-2	cyclooxygenase-2

## References

[1] Siegel R.L., Miller K.D., Jemal A. (2019). Cancer statistics, 2019. CA Cancer J. Clin..

[2] Chhikara B.S., Parang K. Chemical Biology LETTERS Global Cancer Statistics 2022: The Trends Projection Analysis. https://pubs.thesciencein.org/cbl.

[3] Hanahan D., Weinberg R.A. (2011). Hallmarks of cancer: The next generation. Cell.

[4] Fouad Y.A., Aanei C. (2017). Revisiting the hallmarks of cancer. Am. J. Cancer Res..

[5] Ravi S., Alencar A.M., Arakelyan J., Xu W., Stauber R., Wang C.-C.I., Papyan R., Ghazaryan N., Pereira R.M. (2022). An Update to Hallmarks of Cancer. Cureus.

[6] Hanahan D. (2022). Hallmarks of Cancer: New Dimensions. Cancer Discov..

[7] Xiao B., Ma L., Merlin D. (2016). Nanoparticle-mediated co-delivery of chemotherapeutic agent and siRNA for combination cancer therapy. Expert Opin. Drug Deliv..

[8] Abedi-Gaballu F., Dehghan G., Ghaffari M., Yekta R., Abbaspour-Ravasjani S., Baradaran B., Ezzati Nazhad Dolatabadi J., Hamblin M.R. (2018). PAMAM dendrimers as efficient drug and gene delivery nanosystems for cancer therapy. Appl. Mater. Today.

[9] Dilalla V., Chaput G., Williams T., Sultanem K. (2020). Radiotherapy side effects: Integrating a survivorship clinical lens to better serve patients. Curr. Oncol..

[10] Li J., Chen F., Cona M.M., Feng Y., Himmelreich U., Oyen R., Verbruggen A., Ni Y. (2012). A review on various targeted anticancer therapies. Target. Oncol..

[11] Pucci C., Martinelli C., Ciofani G. (2019). Innovative approaches for cancer treatment: Current perspectives and new challenges. Ecancermedicalscience.

[12] van den Boogaard W.M.C., Komninos D.S.J., Vermeij W.P. (2022). Chemotherapy Side-Effects: Not All DNA Damage Is Equal. Cancers.

[13] Sanz Del Olmo N., Carloni R., Bajo A.M., Ortega P., Fattori A., Gómez R., Ottaviani M.F., García-Gallego S., Cangiotti M., De La Mata F.J. (2019). Insight into the antitumor activity of carbosilane Cu(ii)-metallodendrimers through their interaction with biological membrane models. Nanoscale.

[14] Khan A.W., Farooq M., Haseeb M., Choi S. (2022). Role of Plant-Derived Active Constituents in Cancer Treatment and Their Mechanisms of Action. Cells.

[15] Choudhary N., Bawari S., Burcher J.T., Sinha D., Tewari D., Bishayee A. (2023). Targeting Cell Signaling Pathways in Lung Cancer by Bioactive Phytocompounds. Cancers.

[16] Pons D.G. (2024). Roles of Phytochemicals in Cancer Prevention and Therapeutics. Int. J. Mol. Sci..

[17] Jang J.H., Lee T.J. (2023). Mechanisms of Phytochemicals in Anti-Inflammatory and Anti-Cancer. Int. J. Mol. Sci..

[18] Hashem S., Ali T.A., Akhtar S., Nisar S., Sageena G., Ali S., Al-Mannai S., Therachiyil L., Mir R., Elfaki I. (2022). Targeting cancer signaling pathways by natural products: Exploring promising anti-cancer agents. Biomed. Pharmacother..

[19] ELichtenstein M., Sallon S., Paavilainen H., Solowey E., Lorberboum-Galski H. (2014). Evaluating medicinal plants for anticancer activity. Sci. World J..

[20] Talib W.H., Mahasneh A.M. (2010). Antiproliferative activity of plant extracts used against cancer in traditional medicine. Sci. Pharm..

[21] Nelson V.K., Sahoo N.K., Sahu M., Sudhan H.H., Pullaiah C.P., Muralikrishna K.S. (2020). In vitro anticancer activity of *Eclipta alba* whole plant extract on colon cancer cell HCT-116. BMC Complement. Med. Ther..

[22] Gull N., Arshad F., Naikoo G.A., Hassan I.U., Pedram M.Z., Ahmad A., Aljabali A.A.A., Mishra V., Satija S., Charbe N. (2023). Recent Advances in Anticancer Activity of Novel Plant Extracts and Compounds from *Curcuma longa* in Hepatocellular Carcinoma. J. Gastrointest. Cancer.

[23] Garcia-Oliveira P., Otero P., Pereira A.G., Chamorro F., Carpena M., Echave J., Fraga-Corral M., Simal-Gandara J., Prieto M.A. (2021). Status and challenges of plant-anticancer compounds in cancer treatment. Pharmaceuticals.

[24] Nguyen N.H., Hoai Ta Q.T., Pham Q.T., Han Luong T.N., Phung V.T., Duong T.H., Vo V.G. (2020). Anticancer activity of novel plant extracts and compounds from *Adenosma bracteosum* (Bonati) in human lung and liver cancer cells. Molecules.

[25] Greenwell M., Rahman P.K.S.M. (2015). Medicinal Plants: Their Use in Anticancer Treatment. Int. J. Pharm. Sci. Res..

[26] Dixit S., Ali H., Prof A. (2010). Anticancer activity of Medicinal plant extract—A review. J. Chem. Chem. Sci..

[27] Prakash O., Kumar A., Kumar P., Ajeet A. (2013). Anticancer Potential of Plants and Natural Products: A Review. Am. J. Pharmacol. Sci..

[28] Kiraz Y., Adan A., Kartal Yandim M., Baran Y. (2016). Major apoptotic mechanisms and genes involved in apoptosis. Tumour Biol..

[29] Tanos R., Karmali D., Nalluri S., Goldsmith K.C. (2016). Select Bcl-2 antagonism restores chemotherapy sensitivity in high-risk neuroblastoma. BMC Cancer.

[30] Letai A. (2017). Apoptosis and cancer. Annu. Rev. Cancer Biol..

[31] Azab A.E., Azzwali A.-A.A. (2019). Apoptosis: Insight into Stages, Extrinsic and Intrinsic Pathways, Apoptosis: Insight into Stages, Extrinsic and Intrinsic Pathways. Open Sci. J. Clin. Med..

[32] Nair P., Lu M., Petersen S., Ashkenazi A. (2014). Apoptosis initiation through the cell-extrinsic pathway. Methods Enzymol.

[33] Ashkenazi A. (2008). Targeting the extrinsic apoptosis pathway in cancer. Cytokine Growth Factor Rev..

[34] Sayers T.J. (2011). Targeting the extrinsic apoptosis signaling pathway for cancer therapy. Cancer Immunol. Immunother..

[35] Jan R., Chaudhry G.E.S. (2019). Understanding apoptosis and apoptotic pathways targeted cancer therapeutics. Adv. Pharm. Bull..

[36] Rana J.N., Mumtaz S. (2025). Prunin: An Emerging Anticancer Flavonoid. Int. J. Mol. Sci..

[37] Vogler M., Braun Y., Smith V.M., Westhoff M.-A., Pereira R.S., Pieper N.M., Anders M., Callens M., Vervliet T., Abbas M. (2025). The BCL2 family: From apoptosis mechanisms to new advances in targeted therapy. Signal Transduct. Target. Ther..

[38] Fulda S., Debatin K.M. (2006). Extrinsic versus intrinsic apoptosis pathways in anticancer chemotherapy. Oncogene.

[39] Fernández-Luna J.L. (2007). Apoptosis regulators as targets for cancer therapy. Clin. Transl. Oncol..

[40] Papaliagkas V., Anogianaki A., Anogianakis G., Ilonidis G. (2007). The proteins and the mechanisms of apoptosis: A mini-review of the fundamentals. Hippokratia.

[41] Shamas-Din A., Brahmbhatt H., Leber B., Andrews D.W. (2011). BH3-only proteins: Orchestrators of apoptosis. Biochim. Biophys. Acta Mol. Cell Res..

[42] Chota A., George B.P., Abrahamse H. (2021). Interactions of multidomain pro-apoptotic and anti-apoptotic proteins in cancer cell death. Oncotarget.

[43] Silke J., Meier P. (2013). Inhibitorofapoptosis (IAP) proteins-modulators of cell death and inflammation. Cold Spring Harb. Perspect. Biol..

[44] Ola M.S., Nawaz M., Ahsan H. (2011). Role of Bcl-2 family proteins and caspases in the regulation of apoptosis. Mol. Cell Biochem..

[45] Michels J., Johnson P.W.M., Packham G. (2005). Mcl-1. Int. J. Biochem. Cell Biol..

[46] Yip K.W., Reed J.C. (2008). Bcl-2 family proteins and cancer. Oncogene.

[47] Singh R., Letai A., Sarosiek K. (2019). Regulation of apoptosis in health and disease: The balancing act of BCL-2 family proteins. Nat. Rev. Mol. Cell Biol..

[48] Ladokhin A.S. (2020). Regulation of Apoptosis by the Bcl-2 Family of Proteins: Field on a Brink. Cells.

[49] Gregoriou G., Neophytou C.M., Vasincu A., Gregoriou Y., Hadjipakkou H., Pinakoulaki E., Christodoulou M.C., Ioannou G.D., Stavrou I.J., Christou A. (2021). Constantinou, Anti-cancer activity and phenolic content of extracts derived from cypriot carob (*Ceratonia siliqua* L.) pods using different solvents. Molecules.

[50] Ben Ayache S., Saafi E.B., Emhemmed F., Flamini G., Achour L., Muller C.D. (2020). Biological activities of aqueous extracts from carob plant (*Ceratonia siliqua* L.) by antioxidant, analgesic and proapoptotic properties evaluation. Molecules.

[51] Alshammari G.M., Balakrishnan A., Alshatwi A.A., Al-Khalifa A., Zuo B. (2020). Cucurbita ficifolia Fruit Extract Induces Tp53/Caspase-Mediated Apoptosis in MCF-7 Breast Cancer Cells. BioMed Res. Int..

[52] Khan M.A., Singh R., Siddiqui S., Ahmad I., Ahmad R., Upadhyay S., Barkat A., Ali A.M.A., Zia Q., Srivastava A. (2022). Anticancer potential of *Phoenix dactylifera* L. seed extract in human cancer cells and pro-apoptotic effects mediated through caspase-3 dependent pathway in human breast cancer MDA-MB-231 cells: An in vitro and in silico investigation. BMC Complement. Med. Ther..

[53] Aggarwal B., Chandra Bharti A. (2003). Anticancer Potential of Curcumin: Preclinical and Clinical Studies. Anticancer Res..

[54] Deeh P.B.D., Arumugam M., Alagarsamy K., Karanam G., Natesh N.S., Watcho P., Vishwakarma V. (2022). *Phyllanthus muellerianus* and *Ficus exasperata* exhibit anti-proliferative and pro-apoptotic activities in human prostate cancer PC-3 cells by modulating calcium influx and activating caspases. Biologia.

[55] Fouzat A., Hussein O.J., Gupta I., Al-Farsi H.F., Khalil A., Al Moustafa A.E. (2022). *Elaeagnus angustifolia* Plant Extract Induces Apoptosis via P53 and Signal Transducer and Activator of Transcription 3 Signaling Pathways in Triple-Negative Breast Cancer Cells. Front. Nutr..

[56] Haykal T., Younes M., El Khoury M., Ammoury C., Tannous S., Hodroj M.H., Sarkis R., Gasilova N., Menin L., Rizk S. (2021). The pro-apoptotic properties of a phytonutrient rich infusion of *A. cherimola* leaf extract on AML cells. Biomed. Pharmacother..

[57] Ammoury C., Younes M., El Khoury M., Hodroj M.H., Haykal T., Nasr P., Sily M., Taleb R.I., Sarkis R., Khalife R. (2019). The pro-apoptotic effect of a Terpene-rich *Annona cherimola* leaf extract on leukemic cell lines. BMC Complement. Altern. Med..

[58] Dubey A., Yadav P., Peeyush, Verma P., Kumar R. (2022). Investigation of Proapoptotic Potential of *Ipomoea carnea* Leaf Extract on Breast Cancer Cell Line. J. Drug Deliv. Ther..

[59] Khazaei S., Abdul Hamid R., Ramachandran V., Mohd Esa N., Pandurangan A.K., Danazadeh F., Ismail P. (2017). Cytotoxicity and Proapoptotic Effects of *Allium atroviolaceum* Flower Extract by Modulating Cell Cycle Arrest and Caspase-Dependent and p53-Independent Pathway in Breast Cancer Cell Lines. Evidence-Based Complement. Altern. Med..

[60] Abbas Momtazi-Borojeni A., Behbahani M., Sadeghi-aliabadi S.H. (2013). Antiproliferative, Activity and Apoptosis Induction of Crude Extract and Fractions of *Avicennia marina*. Iran. J. Basic Med. Sci..

[61] Badmus J.A., Ekpo O.E., Hussein A.A., Meyer M., Hiss D.C. (2015). Antiproliferative and apoptosis induction potential of the methanolic leaf extract of *Holarrhena floribunda* (G. Don). Evid.-Based Complement. Altern. Med..

[62] Kumar S., Mathew S.O., Aharwal R.P., Tulli H.S., Mohan C.D., Sethi G., Ahn K.-S., Webber K., Sandhu S.S., Bishayee A. (2023). Withaferin A: A Pleiotropic Anticancer Agent from the Indian Medicinal Plant *Withania somnifera* (L.) Dunal. Pharmaceuticals.

[63] Elbouzidi A., Taibi M., Ouassou H., Ouahhoud S., Ou-Yahia D., Loukili E.H., Aherkou M., Mansouri F., Bencheikh N., Laaraj S. (2023). Exploring the Multi-Faceted Potential of Carob (*Ceratonia siliqua* var. Rahma) Leaves from Morocco: A Comprehensive Analysis of Polyphenols Profile, Antimicrobial Activity, Cytotoxicity against Breast Cancer Cell Lines, and Genotoxicity. Pharmaceuticals.

[64] Macuer-Guzmán J., Bernal G., Jamett-Díaz F., Ramírez-Rivera S., Ibáñez C. (2019). Selective and Apoptotic Action of Ethanol Extract of *Annona cherimola* Seeds against Human Stomach Gastric Adenocarcinoma Cell Line AGS. Plant Foods Hum. Nutr..

[65] Lingfa L., Tirumala A., Ankanagari S. (2023). In Vitro Cytotoxicity of Reproductive Stage *Withania somnifera* Leaf and Stem on HepG2 Cell Line. Evidence-Based Complement. Altern. Med..

[66] Feitelson M.A., Arzumanyan A., Kulathinal R.J., Blain S.W., Holcombe R.F., Mahajna J., Marino M., Martinez-Chantar M.L., Nawroth R., Sanchez-Garcia I. (2015). Sustained proliferation in cancer: Mechanisms and novel therapeutic targets. Semin. Cancer Biol..

[67] Zhu J., Thompson C.B. (2019). Metabolic regulation of cell growth and proliferation. Nat. Rev. Mol. Cell Biol..

[68] Schiliro C., Firestein B.L. (2021). Mechanisms of metabolic reprogramming in cancer cells supporting enhanced growth and proliferation. Cells.

[69] Purushotham G., Padma Y., Nabiha Y., Venkata Raju R.R. (2016). In vitro evaluation of anti-proliferative, anti-inflammatory and pro-apoptotic activities of the methanolic extracts of *Andrographis nallamalayana* Ellis on A375 and B16F10 melanoma cell lines. 3 Biotech.

[70] Albinhassan T.H., Saleh K.A., Barhoumi Z., Alshehri M.A., Al-Ghazzawi A.M. (2021). Anticancer, anti-proliferative activity of *Avicennia marina* plant extracts. J. Cancer Res. Ther..

[71] Singh B.N., Shankar S., Srivastava R.K. (2011). Green tea catechin, epigallocatechin-3-gallate (EGCG): Mechanisms, perspectives and clinical applications. Biochem. Pharmacol..

[72] Yang C.S., Wang X., Lu G., Picinich S.C. (2009). Cancer prevention by tea: Animal studies, molecular mechanisms and human relevance. Nat. Rev. Cancer.

[73] Devabattula G., Panda B., Yadav R., Godugu C. (2024). The Potential Pharmacological Effects of Natural Product Withaferin A in Cancer: Opportunities and Challenges for Clinical Translation. Planta Medica.

[74] Lin M., Bi H., Yan Y., Huang W., Zhang G., Zhang G., Tang S., Liu Y., Zhang L., Ma J. (2017). Parthenolide suppresses non-small cell lung cancer GLC-82 cells growth via B-Raf/MAPK/Erk pathway. Oncotarget.

[75] Paul S., Roy D., Pati S., Sa G. (2021). The Adroitness of Andrographolide as a Natural Weapon Against Colorectal Cancer. Front. Pharmacol..

[76] Swargiary A., Roy M.K., Verma A.K. (2021). In vitro study of the antioxidant, antiproliferative, and anthelmintic properties of some medicinal plants of Kokrajhar district, India. J. Parasit. Dis..

[77] Deng X.X., Jiao Y.N., Hao H.F., Xue D., Bai C.C., Han S.Y. (2021). *Taraxacum mongolicum* extract inhibited malignant phenotype of triple-negative breast cancer cells in tumor-associated macrophages microenvironment through suppressing IL-10/STAT3/PD-L1 signaling pathways. J. Ethnopharmacol..

[78] Kheyar N., Bellik Y., Serra A.T., Kheyar F., Bedjou F. (2022). Inula viscosa phenolic extract suppresses colon cancer cell proliferation and ulcerative colitis by modulating oxidative stress biomarkers. BioTechnologia.

[79] D’ANgelo S., Martino E., Ilisso C.P., Bagarolo M.L., Porcelli M., Cacciapuoti G. (2017). Pro-oxidant and pro-apoptotic activity of polyphenol extract from Annurca apple and its underlying mechanisms in human breast cancer cells. Int. J. Oncol..

[80] Rajabi M., Mousa S.A. (2017). The role of angiogenesis in cancer treatment. Biomedicines.

[81] Joga S., Koyyala V.P.B. (2021). Angiogenesis in Cancer. Indian J. Med Paediatr. Oncol..

[82] Nishida N., Yano H., Nishida T., Kamura T., Kojiro M. (2006). Vascular Health and Risk Management ISSN: (Print) (Angiogenesis in Cancer). Vasc. Health Risk Manag..

[83] Liu Z.L., Chen H.H., Zheng L.L., Sun L.P., Shi L. (2023). Angiogenic signaling pathways and anti-angiogenic therapy for cancer. Signal Transduct. Target. Ther..

[84] Kretschmer M., Rüdiger D., Zahler S. (2021). Mechanical aspects of angiogenesis. Cancers.

[85] Al-Ostoot F.H., Salah S., Khamees H.A., Khanum S.A. (2021). Tumor angiogenesis: Current challenges and therapeutic opportunities. Cancer Treat. Res. Commun..

[86] Hoseinkhani Z., Norooznezhad F., Rastegari-Pouyani M., Mansouri K. (2020). Medicinal plants extracts with antiangiogenic activity: Where is the link?. Adv. Pharm. Bull..

[87] Mangir N., Dikici S., Claeyssens F., MacNeil S. (2019). Using ex Ovo Chick Chorioallantoic Membrane (CAM) Assay to Evaluate the Biocompatibility and Angiogenic Response to Biomaterials. ACS Biomater. Sci. Eng..

[88] Sipos S., Moacă E.A., Pavel I.Z., Avram Ş., Crețu O.M., Coricovac D., Racoviceanu R.M., Ghiulai R., Pană R.D., Şoica C.M. (2021). *Melissa officinalis* L. Aqueous extract exerts antioxidant and antiangiogenic effects and improves physiological skin parameters. Molecules.

[89] Magyari-Pavel I.Z., Moacă E.A., Avram Ș., Diaconeasa Z., Haidu D., Ștefănuț M.N., Rostas A.M., Muntean D., Bora L., Badescu B. (2024). Antioxidant Extracts from Greek and Spanish Olive Leaves: Antimicrobial, Anticancer and Antiangiogenic Effects. Antioxidants.

[90] Antoniak K., Studzińska-Sroka E., Szymański M., Dudek-Makuch M., Cielecka-Piontek J., Korybalska K. (2023). Antiangiogenic, Anti-Inflammatory and Antioxidant Properties of *Bidens tripartite* Herb, *Galium verum* Herb and *Rumex hydrolapathum* Root. Molecules.

[91] Kei W.W., Raju N.S.C. (2022). Anti-Angiogenic Screening of Moringa oleifera Leaves Extract Using Chorioallantonic Membrane Assay. Iraqi J. Pharm. Sci..

[92] Muralidharan K., Kumaravelu P., David D. (2021). Evaluation of antiangiogenic and antiproliferative potential of ethanolic extracts of *Andrographis echioides* using in vitro and in ovo assays. J. Cancer Res. Ther..

[93] Niapour A., Miran M., Seyedasli N., Norouzi F. (2022). Anti-angiogenic effects of aqueous extract from *Agrostemma githago* L. seed in human umbilical vein endothelial cells via regulating Notch/VEGF, MMP2/9, ANG2, and VEGFR2. Environ. Sci. Pollut. Res..

[94] Lee J.S., Shukla S., Kim J.A., Kim M. (2015). Anti-angiogenic effect of nelumbo nucifera leaf extracts in human umbilical vein endothelial cells with antioxidant potential. PLoS ONE.

[95] Sajida, Prabhu A. (2021). Anti-angiogenic, apoptotic and matrix metalloproteinase inhibitory activity of *Withania somnifera* (ashwagandha) on lung adenocarcinoma cells. Phytomedicine.

[96] Mohapatra R. (2021). Antigenotoxic and Antiangiogenic Activity of Alternanthera Sessilis, Alstonia Scholaris and Anogeissus Acuminata Leaf Extracts. J. Res. Technol. Educ..

[97] Majeed Al-Shammari A., Razzaq B., Falah Hassan I., Abdul Razzaq Mshimesh B., Mohammad Rasheed A. (2021). Anti-Angiogenic Activity of Annona Reticulata N-Hexane Seeds Extract: In Vivo Study. Artic. Biochem. Cell Arch..

[98] Zhang W., Liu B., Feng Y., Liu J., Ma Z., Zheng J., Xia Q., Ni Y., Li F., Lin R. (2017). Anti-angiogenic activity of water extract from *Euphorbia pekinensis* Rupr. J. Ethnopharmacol..

[99] Kamble S.S., Gacche R.N. (2019). Evaluation of anti-breast cancer, anti-angiogenic and antioxidant properties of selected medicinal plants. Eur. J. Integr. Med..

[100] Serrano-del Valle A., Naval J., Anel A., Marzo I. (2020). Novel Forms of Immunomodulation for Cancer Therapy. Trends Cancer.

[101] Petroni G., Buqué A., Zitvogel L., Kroemer G., Galluzzi L. (2021). Immunomodulation by targeted anticancer agents. Cancer Cell.

[102] Nuzzo G., Senese G., Gallo C., Albiani F., Romano L., D’ippolito G., Manzo E., Fontana A. (2022). Antitumor Potential of Immunomodulatory Natural Products. Mar. Drugs.

[103] Vaghasia H., Patel R., Prajapati J., Shah K., Saraf M., Rawal R.M. (2025). Cytotoxic and immunomodulatory properties of *Tinospora cordifolia*, *Boerhaavia diffusa*, *Berberis aristata*, and *Ocimum basilicum* extracts against HPV-positive cervical cancer cell line. BMC Complement. Med. Ther..

[104] Kis B., Pavel I.Z., Avram S., Moaca E.A., Juan M.H.S., Schwiebs A., Radeke H.H., Muntean D., Diaconeasa Z., Minda D. (2022). Antimicrobial activity, in vitro anticancer effect (MCF-7 breast cancer cell line), antiangiogenic and immunomodulatory potentials of *Populus nigra* L. buds extract. BMC Complement. Med. Ther..

[105] Lemieszek M.K., Komaniecka I., Chojnacki M., Choma A., Rzeski W. (2022). Immunomodulatory Properties of Polysaccharide-Rich Young Green Barley (*Hordeum vulgare*) Extract and Its Structural Characterization. Molecules.

[106] Kashyap V.K., Peasah-Darkwah G., Dhasmana A., Jaggi M., Yallapu M.M., Chauhan S.C. (2022). *Withania somnifera*: Progress towards a Pharmaceutical Agent for Immunomodulation and Cancer Therapeutics. Pharmaceutics.

[107] Li S., Sun Y., Huang J., Wang B., Gong Y., Fang Y., Liu Y., Wang S., Guo Y., Wang H. (2020). Anti-tumor effects and mechanisms of *Astragalus membranaceus* (AM) and its specific immunopotentiation: Status and prospect. J. Ethnopharmacol..

[108] Kim H.R., Kim Y.S., Lee D.R., Choi B.K., Kwon K.B., Bae G.S. (2021). *Echinacea purpurea* alleviates cyclophosphamide-induced immunosuppression in mice. Appl. Sci..

[109] Nurgali K., Jagoe R.T., Abalo R. (2018). Editorial: Adverse effects of cancer chemotherapy: Anything new to improve tolerance and reduce sequelae?. Front. Pharmacol..

[110] Pai V.B., Nahata M.C. (2000). Cardiotoxicity of Chemotherapeutic Agents Incidence, Treatment and Prevention. Drug Saf..

[111] Małyszko J., Kozłowska K., Kozłowski L., Małyszko J. (2016). Nephrotoxicity of anticancer treatment. Nephrol. Dial. Transplant..

[112] M. Publications Pvt Ltd. JCRT_July_08.indd. www.cancerjournal.net.

[113] Tag H.M. (2015). Hepatoprotective effect of mulberry (*Morus nigra*) leaves extract against methotrexate induced hepatotoxicity in male albino rat. BMC Complement. Altern. Med..

[114] Mhatre B.A., Marar T. (2016). Protective effect of *Morinda citrifolia* L. (fruit extract) on methotrexate-induced toxicities—Hematological and biochemical studies. Cogent Biol..

[115] Abouelela M.E., Orabi M.A., Abdelhamid R.A., Abdelkader M.S., Madkor H.R., Darwish F.M., Hatano T., Elsadek B.E.M. (2020). Ethyl acetate extract of *Ceiba pentandra* (L.) Gaertn. reduces methotrexate-induced renal damage in rats via antioxidant, anti-inflammatory, and antiapoptotic actions. J. Tradit. Complement. Med..

[116] LAl Kury L.T., Dayyan F., Shah F.A., Malik Z., Khalil A.A.K., Alattar A., Alshaman R., Ali A., Khan Z. (2020). *Ginkgo biloba* extract Protects against methotrexate-induced hepatotoxicity: A computational and pharmacological approach. Molecules.

[117] Upreti S., Pandey S.C., Bisht I., Samant M. (2021). Evaluation of the target-specific therapeutic potential of herbal compounds for the treatment of cancer. Mol. Divers..

[118] Ghosh S., Das S.K., Sinha K., Ghosh B., Sen K., Ghosh N., Sil P.C. (2024). The Emerging Role of Natural Products in Cancer Treatment. Arch. Toxicol..

[119] Chunarkar-Patil P., Kaleem M., Mishra R., Ray S., Ahmad A., Verma D., Bhayye S., Dubey R., Singh H.N., Kumar S. (2024). Anticancer Drug Discovery Based on Natural Products: From Computational Approaches to Clinical Studies. Biomedicines.

[120] Antunes N., Kundu B., Kundu S.C., Reis R.L., Correlo V. (2022). In Vitro Cancer Models: A Closer Look at Limitations on Translation. Bioengineering.

[121] Sarnella A., Ferrara Y., Terlizzi C., Albanese S., Monti S., Licenziato L., Mancini M. (2024). The Chicken Embryo: An Old but Promising Model for In Vivo Preclinical Research. Biomedicines.

[122] Bokhout L., Campeiro J.D., Dalm S.U. (2025). Exploring the landscape of current in vitro and in vivo models and their relevance for targeted radionuclide theranostics. Eur. J. Nucl. Med..

[123] Han B., Kim S.-M., Nam G.E., Kim S.-H., Park S.-J., Park Y.-K., Baik H.W. (2020). A Randomized, Double-Blind, Placebo-Controlled, Multi-Centered Clinical Study to Evaluate the Efficacy and Safety of *Artemisia annua* L. Extract for Improvement of Liver Function. Clin. Nutr. Res..

[124] Zoi V., Galani V., Lianos G.D., Voulgaris S., Kyritsis A.P., Alexiou G.A. (2021). The role of curcumin in cancer treatment. Biomedicines.

[125] Ameer S.F., Mohamed M.Y., Elzubair Q.A., Sharif E.A.M., Ibrahim W.N. (2024). Curcumin as a novel therapeutic candidate for cancer: Can this natural compound revolutionize cancer treatment?. Front. Oncol..

[126] Anand P., Kunnumakkara A.B., Newman R.A., Aggarwal B.B. (2007). Bioavailability of curcumin: Problems and promises. Mol. Pharm..

[127] Nelson K.M., Dahlin J.L., Bisson J., Graham J., Pauli G.F., Walters M.A. (2017). The Essential Medicinal Chemistry of Curcumin. J. Med. Chem..

[128] Pavese J.M., Krishna S.N., Bergan R.C. (2014). Genistein inhibits human prostate cancer cell detachment, invasion, and metastasis. Am. J. Clin. Nutr..

[129] Ballesteros-Ramírez R., Pinilla P., Sanchéz J., Torregrosa L., Aschner P., Urueña C., Fiorentino S. (2023). Safety and efficacy of P2Et extract from *Caesalpinia spinosa* in breast cancer patients: Study protocol for a randomized double blind phase II clinical trial (CS003-BC). BMC Complement. Med. Ther..

[130] Duran M.I., Ballesteros-Ramírez R., Tellez A., Torregrosa L., Olejua P.A., Galvis S., Urueña C., Fiorentino S. (2022). Safety Evaluation in Healthy Colombian Volunteers of P2Et Extract Obtained from *Caesalpinia spinosa*: Design 3+3 Phase i Clinical Trial. Evid.-Based Complement. Altern. Med..

[131] Wagner H., Ulrich-Merzenich G. (2009). Synergy research: Approaching a new generation of phytopharmaceuticals. Phytomedicine.

[132] Liu R.H. (2004). Potential Synergy of Phytochemicals in Cancer Prevention: Mechanism of Action 1. J. Nutr..

[133] Newman D.J., Cragg G.M. (2020). Natural Products as Sources of New Drugs over the Nearly Four Decades from 01/1981 to 09/2019. J. Nat. Prod..

[134] Bozzuto G., Calcabrini A., Colone M., Condello M., Dupuis M.L., Pellegrini E., Stringaro A. (2024). Phytocompounds and Nanoformulations for Anticancer Therapy: A Review. Molecules.

[135] Ali Abdalla Y.O., Subramaniam B., Nyamathulla S., Shamsuddin N., Arshad N.M., Mun K.S., Awang K., Nagoor N.H. (2022). Natural Products for Cancer Therapy: A Review of Their Mechanism of Actions and Toxicity in the Past Decade. J. Trop. Med..

